# Antecedents of Psychological Contract Breach: The Role of Job Demands, Job Resources, and Affect

**DOI:** 10.1371/journal.pone.0154696

**Published:** 2016-05-12

**Authors:** Tim Vantilborgh, Jemima Bidee, Roland Pepermans, Yannick Griep, Joeri Hofmans

**Affiliations:** 1 Department of Experimental and Applied Psychology, Work and Organizational Psychology research unit, Vrije Universiteit Brussel, Pleinlaan 2, 1050 Brussel, Belgium; 2 Department of Psychology, University of Calgary, 2500 University Drive, Northwest Calgary, AB T2N 1N4, Canada; Liverpool School of Tropical Medicine, UNITED KINGDOM

## Abstract

While it has been shown that psychological contract breach leads to detrimental outcomes, relatively little is known about factors leading to perceptions of breach. We examine if job demands and resources predict breach perceptions. We argue that perceiving high demands elicits negative affect, while perceiving high resources stimulates positive affect. Positive and negative affect, in turn, influence the likelihood that psychological contract breaches are perceived. We conducted two experience sampling studies to test our hypotheses: the first using daily surveys in a sample of volunteers, the second using weekly surveys in samples of volunteers and paid employees. Our results confirm that job demands and resources are associated with negative and positive affect respectively. Mediation analyses revealed that people who experienced high job resources were less likely to report psychological contract breach, because they experienced high levels of positive affect. The mediating role of negative affect was more complex, as it increased the likelihood to perceive psychological contract breach, but only in the short-term.

## Introduction

Whenever someone enters an exchange agreement, a psychological contract (PC) is formed. This PC contains the person’s perception of the mutual obligations between both parties involved in the exchange [1] and therefore PCs are critical to understand the relationship between employees and employers. Over two decades of research has firmly established that PC breach—or the awareness that the employer fails to fulfill one or more obligations included in the PC—has negative consequences such as reduced job satisfaction, organizational commitment, performance, and increased turnover intentions [2,3]. While there is an abundance of research on the consequences of PC breach, less is known about its antecedents [4]. This paper addresses this gap in the PC literature by introducing two potential antecedents of PC breach: (1) job demands and resources and (2) affect. In the current study we focus on state affect. State affect needs to be conceptually distinguished from emotions. Whereas emotions are relatively short-lived and involve a specific cause, state affect is more long-lasting and objectless [49]. We nonetheless sometimes refer to emotions when describing findings of previous studies because we use the authors’ original wordings. Based on the Job Demands-Resources model [5] and the Affect Infusion Model [6], we propose that people who experience high demands and/or few resources are more likely to perceive PC breach because they experience a decrease in positive affect and an increase in negative affect.

This paper contributes to the literature in important ways. First, it studies the factors leading to perceptions of PC breach. Conway and Briner [7] emphasized that there is an important gap in the literature with regards to understanding the processes underlying the formation of PCs in general and of PC breach in particular. So far, studies have demonstrated that factors such as trust in the employer [8], personality [9], and human resources practices [10] shape PC breach perceptions. We extend this stream of research and argue that situational characteristics—i.e., experienced job demands and resources—can increase or decrease the probability that one perceives that the organization is not fulfilling its end of the deal. We further argue that affect plays a central role in this process, suggesting that changes in experienced job demands and resources result in changes in positive and negative affect, which in turn influence the likelihood of perceiving PC breach. Hence, we do not merely treat affect as a by-product of PC breach, but also as a source of information used by employees to inform them about the state of their PC.

Second, a growing body of research shows that perceptions of PC breach vary on a weekly or even daily basis, and that these within-person fluctuations predict a substantial amount of within-person variation in emotions, counterproductive work behavior, and organizational commitment [11–13]. Despite these recent advances, most studies have examined antecedents and consequences of PC breach using cross-sectional designs or via longitudinal designs with large time-intervals [7]. As a result, these studies are unable to capture the within-person processes underlying PC breach. In the present study, we examine such within-person processes by repeatedly surveying the same participants as they go about their daily lives (i.e., daily measurements in Study 1, weekly measurements in Study 2) [14]. This is an important contribution to the PC literature as studying within-person processes has been put forward as one of the major challenges for contemporary PC research [7].

In what follows, we first introduce the psychological contract literature. Next, we explain how job demands and resources influence PC breach perceptions, via affect. Finally, we present findings from two experience sampling studies on these antecedents of PC breach and discuss implications for theory and practice.

### The psychological contract

Psychological contracts have been defined as “*an individual’s beliefs regarding the terms and conditions of a reciprocal exchange agreement between that focal person and another party*” [1]. Most research focuses on the consequences of PC breach or an individual’s perception that the organization fails to meet it’s obligations towards that individual [15].

According to Morrison and Robinson [15], people may perceive that an obligation is breached because the organization is unable or unwilling to deliver the obligated inducement (*reneging*) or because the individual and the organization have divergent perceptions about mutual obligations (*incongruence*). Moreover, Morrison and Robinson [15] argue that not every unmet obligation will result in a PC breach because individuals consider the ratio of inducements that are provided by the organization to the inducements that are obligated by the organization. A PC breach will only be noticed once a threshold in this ratio is exceeded. As a result, a minor deviation from an obligation may go by unnoticed.

O’Neill, Halbesleben, and Edwards [16] provide an alternative view on antecedents of PC breach, based on Goodman’s [17] three categories of social comparisons. They argue that PC breach occurs because people compare workplace occurrences to oneself (e.g. past experiences; *self-referent comparisons*), to others (e.g. colleagues’ experiences; *other-referent comparisons*), and to environmental elements (e.g. organization’s mission statement; *structural triggers*). These comparisons cause people to evaluate their PC, and therefore they determine the likelihood that PC breach will be perceived.

We propose that job demands and resources are related to perceptions of PC breach because they trigger self-referent comparisons [16]. Individuals compare the demands placed on them and the resources offered to them to their past experiences. For example, a person who typically works 40 hours per week but is suddenly asked to increase this engagement to 45 hours per week, may decide that this is not in line with past experiences and, hence, that this is not part of the deal. Moreover, we propose that this effect is not a direct one, but that job demands and resources influence perceptions of PC breach indirectly through the comparison process that is triggered by unmet expectations [15]. In particular, we argue that perceptions of job demands and resources are accompanied by positive and negative affect, which in turn bias the comparison process: a person experiencing high negative and/or low positive affect is more likely to interpret an unmet obligation as a PC breach [15]. In sum, we propose that job demands and resources are indirectly related to perceptions of breach via positive and negative affect.

### Relating job demands and resources to affect

According to the Job Demands-Resources (JD-R) model, job characteristics can be divided into demands and resources [5,18]. Job demands (e.g. high workload, emotional demands) are “*physical*, *psychological*, *social*, *or organisational aspects of the job that require sustained physical and/or psychological (cognitive and emotional) effort or skills and are therefore associated with certain physiological and/or psychological costs*” [5]. In contrast, job resources (e.g. social support, autonomy) are physical, psychological, social, or organizational aspects of the job that (1) help the individual to achieve work goals, (2) reduce job demands and (3) stimulate personal growth, learning, and development [5]. The JD-R model proposes that higher job demands trigger a health-impairment process that gradually depletes the individual’s energy and causes health problems because of the required sustained effort. In contrast, more job resources trigger a motivational process, leading to high work engagement, low cynicism, and effective performance.

We propose that job demands and resources relate to experiences of positive and negative state affect. People who experience high positive state affect feel enthusiastic, active, and alert [19]. A high positive affective state is characterized by “high energy, full concentration, and pleasurable engagement” [19]. People who experience high negative state affect feel a variety of negative mood states, such as anger, contempt, disgust, fear, and nervousness [19].

From a theoretical point of view, both job demands and resources are believed to have a motivational potential: resources help to satisfy basic needs, while demands hinder need satisfaction [20]. Satisfying one’s needs in turn triggers positive emotions while hindered needs trigger negative emotions [21]. Therefore, high levels of job resources should lead to positive affect while high levels of job demands are expected to lead to negative affect. From an empirical point of view, the relationships between job demands and resources and positive-negative affect received support in a number of studies. For example, high daily levels of job resources have been associated with high daily levels of positive emotions [22] and of enjoyment [23]. Likewise, job demands were found to relate to variables tapping into employee ill-being, such as burnout and depression [24,25]. Similarly, Zohar [26] reported that encountering daily hassles at work—akin to job demands—predicted negative mood at the end of the day. In sum, we propose that:

**Hypothesis 1:** *High levels of job resources are associated with high levels of positive affect*.

**Hypothesis 2:** *High levels of job demands are associated with high levels of negative affect*.

### Affect as an antecedent of psychological contract breach

The Affect Infusion Model (AIM) offers a theoretical rationale for affect as an antecedent of PC breach [6,27]. Affect infusion is “*the process whereby affectively loaded information exerts an influence on and becomes incorporated into the judgmental process*, *entering into the judge’s deliberations and eventually coloring the judgmental outcome*” [6]. First, affect can influence judgments because people consider it a piece of information. For example, persons reflecting on past events may judge that their PC was breached because they use their negative affective state to signal that something is wrong in the exchange agreement. Second, affect can influence judgments via affect priming, meaning that it causes selective attention, encoding, and retrieval of information, and directs associations and interpretations. For example, persons who ruminate over past events are more likely to decide that their PC is breached, because their negative affective state translates into one paying more attention to negative occurrences that week. Reflecting on whether or not one’s PC was breached requires open thinking and interpreting ambiguous situations with incomplete information, and is therefore likely infused by affect [6].

As mentioned above, a couple of studies examined affect as an outcome of PC breach [13,28,29]. To our knowledge, no study to date has studied whether affect also precedes PC breach. Nonetheless, Morrison and Robinson [15], as well as more recent theorizing on psychological contract theory [30], proposed that affect could influence the likelihood to perceive PC breaches. In particular, positive affect is believed to downplay discrepancies and increase the likelihood that environmental cues are interpreted as congruent with the PC, while negative affect stimulates people to monitor their environment for cues signaling discrepancies in their PC [30]. In sum, there are theoretical arguments based on the AIM [6] and psychological contract theory [15,30] suggesting that positive/negative affect relates to PC breach perceptions. We therefore propose that affect acts as an antecedent of PC breach, with:

**Hypothesis 3:** *High levels of positive affect are associated with lower levels of PC breach*.

**Hypothesis 4:** *High levels of negative affect are associated with higher levels of PC breach*.

Because we already argued that perceptions of job demands and resources relate to negative and positive affect respectively, a causal chain or mediation model emerges. Perceptions of working conditions can sensitize individuals to perceive a breach in their PC, because they alter the individual’s affective state. When persons notice many demands / few resources in their environment, they will experience more negative / less positive affect, respectively. These changes in affective state in turn color the individuals’ evaluation of their PC, increasing the likelihood that they will conclude that the organization is breaching one or more obligations in the PC. Combining the above arguments results in an indirect effect of job demands and resources on perceptions of PC breach, via positive and negative affect. This mediation model aligns theoretically with Affective Events Theory [31]. According to this theory, the workplace is characterized by positive and negative affective events (e.g., high job demands such as a cognitively demanding task or high job resources such as coworkers offering emotional support during a stressful day). These affective events generate affective states, or momentary fluctuations in positive and negative affect, which in turn shape the attitudes or perceptions about the workplace relationship (e.g., commitment) or perceptions of the extent to which the organization fulfills the PC. Building on Affective Events Theory [31], we propose that job demands and resources are indirectly related to perceptions of PC breach, via an affective pathway.

**Hypothesis 5:** *The relationship between job demands and PC breach is mediated by negative affect*.

**Hypothesis 6:** *The relationship between job resources and PC breach is mediated by positive affect*.

We tested the antecedents of PC breach with volunteering samples. Volunteers—defined as people who freely engage in productive activities to the benefit of others outside of the extended household, without receiving any remuneration [32]—form “*a particularly interesting group to study in terms of the psychological contract because they are not bound to the organization they ‘work’ for by the usual ties of employment*” [33]. In other words, due to the lack of a formal contract, the PC plays an important role in the exchange agreement between volunteers and organizations. Recent studies have indeed shown that the PC is important in a volunteering context and that PC breaches have negative consequences for volunteer wellbeing and performance [11,34,35].

## Study 1

### Method

#### Research context

Study 1 took place in a large Belgian youth organization (>80000 members and >16000 volunteers), offering weekly activities—typically taking place on Sunday—for children and adolescents. Volunteers—often young (between 18 and 26 years) and former members of the organization—organize the activities. The organization is divided into 938 local groups who operate independently and who receive support from the organization’s national administration.

#### Sample and procedure

The volunteer manager of the organization agreed to have about ten local groups participating in the study. After launching an organization-wide call, the volunteer manager provided us with a list of ten groups who were willing to participate in the study. One local group dropped out after being contacted by the authors. The first and second author explained the purpose and procedure of the study during a meeting with the remaining nine local group’s volunteers and administered 96 pen-and-paper general surveys to the meeting attendants. This general survey was used to measure demographic variables. Meeting attendants also received a bundle containing ten pen-and-paper copies of the daily survey. They were instructed to complete one copy at the end of each day on which they had volunteered for the organization. Completed surveys could be directly returned to the researchers in a pre-stamped envelope. Because some volunteers were absent at the aforementioned meetings, general surveys and daily surveys were also distributed via e-mail as online surveys to 56 volunteers. These e-mails contained detailed instructions on the purpose and procedure of the study. To ensure quality of data, confidentiality was guaranteed to all participants. Moreover, participants were instructed to only complete the daily survey on the same day that they volunteered and were asked to indicate the date of completion on each pen-and-paper survey. Electronic surveys were automatically time-stamped. To increase response rates, two gift certificates were raffled.

In total, 146 of the 152 volunteers who attended meetings or were contacted electronically returned a completed general survey (*N*pen−and−paper = 95; *N*electronic = 51). 51 volunteers completed and returned daily surveys (*N*pen−and−paper = 22; *N*electronic = 29). Our final sample consisted of 42 respondents (response rate = 27.36% of the 152 contacted volunteers) as we only retained respondents who had minimally completed three daily surveys, yielding an effective sample size of 289 completed daily surveys or data points (response rate = 19.01% out of a maximum of 1520 daily surveys that were distributed to the 152 contacted volunteers). A minimum of three responses per respondent ensured that we had adequate power to detect medium and large fixed effects on the within-person level [55]. All subsequent references to respondents refer to this final sample.

On average, respondents returned 6.88 (*SD* = 2.69) daily surveys, spanning a period of 3.92 weeks (*SD* = 3.12). Our sample consisted mainly of female volunteers (64.29%), while respondents’ average age was 20.64 years (*SD* = 5.05, range = 18–51). Most respondents had maximally attained a high school degree (88.10%), and only few had attained bachelor degrees (11.90%). Besides volunteering, the majority of the respondents were enrolled as students (90.48%), while the remainder worked as paid employees (9.52%). On average, respondents had volunteered for 2.61 years (*SD* = 3.77) and volunteered 30.86 hours per month (*SD* = 9.72).

#### Ethics statement

Study 1 was exempted from ethical approval by the Medical Ethics Committee of the authors’ university (2014/339), because the research design was deemed non-invasive and harmless. The participating organization first asked local groups of volunteers whether they were willing to participate in the study and if this was the case, they provided us with the local volunteer coordinators’ contact details. Next, the first or second author visited the local group to explain the purpose of the study, stressing the discretionary nature of participation, the possibility to withdraw from the study, and the confidential treatment of the data. This explanation was also provided in writing to participants, meaning that an oral and written informed consent was offered. The authors had access to contact details of participants who completed online daily surveys (i.e., email addresses) to send the questionnaires to the participants and to link the repeated measurements of the same individual. Data were fully anonymized prior to the analyses. Participants who completed the pen-and-paper version of the daily surveys were given an individual code to link repeated measurements of the same individual.

#### Measures

We measured positive and negative affect in the daily surveys with the nine-item Emmons Mood Indicator [36]. Positive affect was measured with four items (joyful, happiness, pleased, and enjoyment) and negative affect with five items (worried/anxious, depressed, frustrated, angry/hostile, and unhappy). Respondents indicated to what extent they had experienced each emotion while volunteering that day on a seven-point rating scale ranging from 1 (*not at all*) to 7 (*extremely*). Cronbach Alpha scores—calculated for each measurement moment separately—indicated that the internal reliabilities of the positive affect (*M*(α) = .93, *SD*(α) = .02, range (α) = .90 to .96) and the negative affect measures (*M*(α) = .79, *SD*(α) = .06, range (α) = .71 to .85) were satisfactory.

We measured job demands and resources in the daily surveys using items from Butler, Grzywacz, Bass, and Linney [37], adapted for a volunteering context (i.e., “work” was reformulated as “voluntary activities”). Job demands were measured with a single item (“I had too many demands on me during my voluntary activities today”), and job resources with two items that focus on the level of control experienced (e.g. “I had a say in deciding what tasks I did during my voluntary activities today”; *M(r)* = .76, *SD(r)* = .13, range (*r*) = .61 to .94). Respondents indicated to what extent they had experienced demands and resources that day on a seven-point rating scale, ranging from 1 (*Totally disagree*) to 7 (*Totally agree*). While short or single-item scales have been criticized for low reliability, Fisher and To [14] argue that surveys in experience sampling studies should be short to minimize burden on respondents. Moreover, they state that abbreviated or single-item scales are acceptable if they have face- and content-validity, and if they correlate with other variables as they should. Cross-validation in Study 2 (see S2 Appendix) confirmed that the single-item job demands measure (*r* = .57, *p* < .001) and the two-item job resources measure (*r* = .45, *p* < .001) correlated significantly and in the expected direction with longer, validated scales [38]. In addition, Butler and colleagues [37] also demonstrated that these short scales are valid measures of job demands and resources in experience sampling research.

We used a single item to measure PC breach in the daily surveys (see S1 Appendix). Following prior studies [8,39], we used a direct measure of PC breach. In particular, we presented respondents with a 0–100 graphical rating scale (i.e., a line of 10 cm), thereby following Fisher and To’s [14] recommendation to use a large number of response options when single item measures are used in daily studies. Respondents indicated on the graphical scale to what extent the organization had fulfilled its obligations to the volunteer that day. This graphical scale had three anchors (far more fulfilled than expected [at 0 cm]; exactly fulfilled as expected [at 5 cm]; far less fulfilled than expected [at 10 cm]). Consequently, the higher the score on this scale, the greater the magnitude of PC breach experienced that particular day. Cross-validation in Study 2 (see S2 Appendix) confirmed that the single-item PC breach measure (*r* = .38, *p* < .001) correlated significantly and in the expected direction with a longer, validated scale [40].

We used the general survey to collect demographic information on respondents’ age (in years), gender (female or male), educational background (highest level of formal education), occupational background, average hours volunteered per month, and company tenure (in years).

### Results and Discussion

#### Confirmatory factor analysis

As a first step, we tested whether job demands and resources, positive and negative affect, and PC breach can be empirically distinguished from each other. To this end, we performed a series of multilevel confirmatory factor analyses (CFAs) in Mplus version 7 [41]. In each of these models, we specified the same factor structure at the within- and between-person level. Model fit was evaluated using the Root Mean Square Error of Approximation (.05 < RMSEA ≤ .08: reasonable fit; 0 ≤ RMSEA ≤ .05: close fit), the Comparative Fit Index (.90 ≤ CFI < .95: good fit; .95≤ CFI ≤ 1.00: excellent fit), and the Tucker-Lewis Index (.90 ≤ TLI < .95: good fit; .95≤ TLI ≤ 1.00: excellent fit) [42]. Competing models were compared using loglikelihood ratio tests. A more detailed discussion of the analyses is provided in S3 Appendix.

We started by estimating a model that matched the theory-based factor structure by including five latent factors for PC breach, job demands, job resources, positive affect, and negative affect. This theory-based model fitted the data well (see Table 1), with each item loading significantly onto its respective latent factors. Next, we estimated three alternative models. Alternative model A included four latent factors for job resources, positive affect, negative affect, and the combination of job demands and PC breach. Alternative model A fitted equally well to the data as the theory-based model (Δχ^2^(6) = 2.26, *p* = .89), but was rejected because not all items loaded significantly on their latent factor. Alternative model B included four latent factors for job demands, positive affect, negative affect, and the combination of job resources and PC breach. Alternative model B fitted significantly worse to the data than the theory-based model (Δχ^2^(8) = 162.57, *p* < .001). Alternative model C included four latent factors for PC breach, job demands, job resources, and the combination of positive and negative affect. Alternative model C fitted significantly worse to the data than the theory-based model (Δχ^2^(9) = 151.00, *p* < .001).

**Table 1:** Results from confirmatory factor analyses in study 1.

Model	*χ*^*2*^ *(df)*	RMSEA	CFI	TLI
Theory-based model	263.34 (128)	.06	.93	.91
Alternative model A	262.95 (134)	.06	.93	.92
Alternative model B	485.28 (136)	.09	.81	.78
Alternative model C	578.29 (137)	.11	.76	.73
CMV model–constrained	260.11 (126)	.06	.93	.91
CMV model—unconstrained	254.01 (115)	.07	.92	.90

*Notes*. *N (within) =* 288, *N (between)* = 42.

*Theory-based model*: *Five latent factors = PC breach*, *job demands*, *job resources*, *positive affect*, *negative affect*. *Alternative model A*: *Four latent factors = PC breach + job demands*, *job resources*, *positive affect*, *negative affect*. *Alternative model B*: *Four latent factors = job demands*, *PC breach + job resources*, *positive affect*, *negative affect*. *Alternative model C*: *Four latent factors = PC breach*, *job demands*, *job resources*, *positive + negative affect*. *CMV model–constrained*: *Six latent factors = PC breach*, *job demands*, *job resources*, *positive affect*, *negative affect*, *common method factor (all factor loadings constrained to be equal) CMV model–unconstrained*: *Six latent factors = PC breach*, *job demands*, *job resources*, *positive affect*, *negative affect*, *common method factor (all factor loadings freely estimated)*.

Next, the presence of common method variance was assessed by testing a model in which we added a latent common method factor to the theory-based model [43]. Next to their factor loadings to one of the other five latent factors (i.e., PC breach, job demands, job resources, positive affect, and negative affect), all indicators in the model also loaded on a latent common method factor. In the constrained CMV model, the factor loadings to the latent common method factor were all constrained to be equal, meaning that common method variance impacted responses to all items in a similar way. The fit of this constrained CMV model was not significantly better than the fit of the theory-based model (Δχ^2^(2) = 3.80, *p* = .15). In the unconstrained CMV model, the factor loadings to the latent common method factor were freely estimated, meaning that the effect of common method variance could differ between the indicators. The unconstrained CMV model also did not offer a better fit to the data than the theory-based model (Δχ^2^(13) = 21.77, *p* = .06). These tests suggest that common method variance has not substantively influenced the responses.

#### Multilevel path model

The Intraclass Correlation Coefficient (ICC) values of positive affect (.16), negative affect (.27), and PC breach (.15) indicated that there was substantial variance at the between-person level. To account for these between-person differences, we tested our hypotheses using multilevel path modeling. As we were only interested in within-person relationships, the independent variables were person-mean centered prior to testing the model. Person-mean centering a variable implies that the respondent’s average score on that variable across the different measurement moments is subtracted from that respondent’s score on the same variable for each measurement moment. As a consequence, all between-person variance is removed from the data. In addition, person-mean centering aligns with social comparison theory and self-referent comparisons as person-mean centered variables express a respondent’s level on a variable as a deviation from that person’s typical (i.e., average) experience.

We started by comparing a full (i.e., the relationship between job demands/resources and PC breach is fully accounted for by positive and negative affect) to a partial mediation model (i.e., the relationship between job demands/resources and PC breach is partially accounted for by positive and negative affect). Because the partial mediation model was just-identified (i.e., no degrees of freedom left), we compared models by looking at the Bayesian Information Criterion (BIC). Smaller BIC values indicate better model fit. This comparison revealed that the partial mediation model fitted the data (*χ*^*2*^(0) = 0, RMSEA = 0, CFI = 1, TLI = 1, BIC = 3543.23) better than the full mediation model (*χ*^*2*^(2) = 10.68, RMSEA = .12, CFI = .95, TLI = .76, BIC = 3543.92).

**Fig 1:**
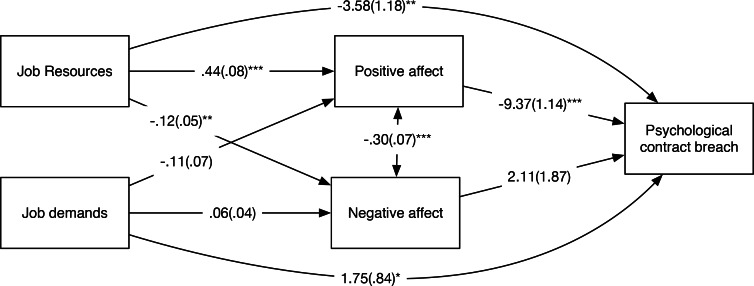
Path estimates from model with concurrent relationships in Study 1. *: *p* < .05, **: *p* < .01, ***: *p* < .001. Standard errors between parentheses.

In this partial mediation model (see Fig 1), job resources related positively to positive affect (γ = .44, *p* < .001). This indicates that volunteers reported higher levels of positive affect on days when they also perceived high job resources, which is in line with Hypothesis 1. There was no significant relationship between job demands and negative affect (γ = .06, *p* = .12), disconfirming Hypothesis 2. However, negative affect was negatively related with job resources (γ = -.12, *p* = .01), meaning that volunteers reported lower levels of negative affect on days that they perceived a high amount of job resources. There was a significant negative relationship between PC breach and positive affect (β = -9.37, *p* < .001), but not between PC breach and negative affect (β = 2.11, *p* = .27). This offered support for Hypothesis 3, but not for Hypothesis 4. Put differently, volunteers reported lower levels of PC breach on days that they experienced high levels of positive affect. In line with the idea of partial mediation, job demands (γ = 1.75, *p* = .04) and job resources (γ = -3.58, *p* = .002) had unique direct effects on perceptions of PC breach after accounting for the mediation through affect.

To assess Hypothesis 5 and 6, we estimated indirect (or mediation) effects using the product-of-coefficients approach [44]. There was a significant indirect effect of job resources on psychological contract breach via positive affect (*estimate* = -4.08, *p* < .001). In other words, on days that volunteers perceived high levels of job resources they reported low levels of psychological contract breach because they experienced high positive affect that day. The indirect effect of job demands on psychological contract breach via negative affect was not significant (*estimate* = -.12, *p* = .40). Hence, these findings support Hypothesis 5, but reject Hypothesis 6.

In summary, the results of our first study offered mixed support for our hypotheses. We found that higher levels of positive affect were reported on days that high job resources were perceived, and that experiencing positive affect decreased the likelihood to report PC breach. As such, our findings supported a mediation effect through positive affect. At the same time, we did not find a similar effect for negative affect. Job demands were unrelated to negative affect, and negative affect did not increase the likelihood to report PC breach. Although the divergence between our findings and previous findings might be due to the fact that previous studies did not control for the shared variance between job demands and job resources and focused on variables tapping into employee well- or ill-being, such as burnout and depression, rather than negative affect, the results might also be due to the specifics of our study.

One important specific is that we studied volunteers while other studies have predominantly focused on paid employees. Although recent studies show that—akin to paid employees—volunteers perceive mutual obligations between themselves and their organization [45,46] and respond negatively to PC breach [11,34,35], there are also subtle differences between both groups’ PCs in terms of content—e.g., volunteers do not perceive any obligations related to pay [46]—and importance of the PC in the exchange agreement. To make sure that the choice to focus on volunteers did not affect our findings, we conducted a second study in which we studied both volunteers and paid employees. At the same time, we also tackled a number of other limitations of Study 1. Because of this reason, Study 2 can be considered a conceptual replication-plus-extension of Study 1, meaning that the operationalization and sample differs and that we add new elements to the design of Study 1. In what follows, we will discuss how Study 2 addresses important limitations of Study 1.

In Study 1, we were unable to make strong claims about temporal precedence as we tested concurrent relationships, or relationships between variables measured on the same day. The use of time-lagged variables was not possible because the time-lag between surveys varied both within and between respondents. In Study 2 we will resolve this issue by testing both concurrent (relationship between independent variable measured at time T and dependent variable measured at time T) and time-lagged (relationship between independent variable measured at time T-1 and dependent variable measured at time T) relationships.

Additionally, Study 1 focused on general demands and resources. In Study 2 we will zoom in on specific demands (cognitive load and work load) and resources (social support and autonomy). By doing so, we can test if the proposed relationships are sensitive to unique characteristics of specific demands and resources.

Finally, we did not measure the extent to which job demands and resources were expected by volunteers. One might argue that the relationships between job demands and negative affect and between job resources and positive affect become weaker if people already expect high levels of job demands and resources. Study two tackles this limitation by measuring the extent to which individuals expect job demands and resources, next to weekly measures of perceived levels of job demands and resources. This enables us to check if expected job demands and resources moderate the relationships between perceived job demands and resources on the one hand and affect on the other hand.

## Study 2

### Method

#### Research context

Study 2 took place in three Belgian social profit enterprises, each consisting of both volunteers and paid staff. Organization A is a large Belgian non-governmental organization focusing on third-world aid. They count over 15000 volunteers, who are divided into 330 local groups and managed by 58 paid employees. Volunteer tasks include acquiring funds, communicating with donors, and campaigning. Organization B is a medium-sized nonprofit organization focusing on youth information. They rely on 37 paid employees and 60 volunteers, who organize workshops on youth issues, social and cultural guidance in youth work, and communication. Organization C is a small nonprofit organization focusing on performing arts. They count 19 paid staff members and 20 to 40 volunteers, depending on their ongoing projects. Volunteer tasks include cleaning, selling tickets, and preparing stages for shows.

#### Sample and procedure

The volunteer coordinator in each organization forwarded an e-mail explaining the purpose of the study and including a link to a general online survey—measuring personal characteristics and demographic variables—to its paid staff and volunteers. Organization A’s volunteer coordinator allowed us to contact 10 out of 330 local groups, because this organization had a very large pool of volunteers of which part were already partaking in another study. General survey respondents could indicate if they were willing to participate in the weekly survey study. Those who agreed were sent an individualized e-mail each Friday at 11AM during the following five weeks containing a link to the online weekly survey. This weekly survey could be completed until Sunday 11PM that same week and surveyed the weekly survey respondents’ perceptions with regards to job demands and resources, positive and negative affect, and PC breach that week. If weekly survey respondents did not work or volunteer during a particular week, the measurements for that week were treated as missing.

In total, 119 out of 700 contacted volunteers and paid employees completed the general survey (*N*_*organization A*_ = 65, *N*_*organization B*_ = 38, *N*_*organization C*_ = 16). 109 volunteers and paid employees also completed the weekly surveys (*N*_*organization A*_ = 64, *N*_*organization B*_ = 32, *N*_*organization C*_ = 13). Hence, our final sample contained 109 individuals (response rate = 15.57% of the contacted volunteers and paid employees), who completed 316 weekly surveys (on average, 2.9 completed weekly surveys per respondent; response rate = 9.03% out of a maximum of 3500 weekly surveys that were distributed to the 700 contacted volunteers and paid employees). All subsequent references to respondents refer to this final sample.

Slightly more than half of the respondents were volunteers (55.32%), while the remaining participants were paid employees (44.68%). Respondents, on average, were 40.51 years old (*SD* = 14.90, range = 17–73) and had 13.76 years tenure (*SD* = 11.98). Slightly more than half of the respondents were female (56.38%); most respondents held a university (55.32%) or college (24.47%) degree and the remainder held a secondary school degree (19.15%) or lower (1.06%). Volunteers spent, on average, 12.14 hours per month (*SD* = 16.31) volunteering.

#### Ethics statement

Study 2 was exempted from ethical approval by the Medical Ethics Committee of the authors’ university (2014/339), because the research techniques were considered non-invasive and harmless. Participating organizations contacted volunteers and paid employees by e-mail, asking them whether they were willing to participate in the study. Respondents who agreed to participate could directly proceed to the general online survey via a link in the e-mail. At the end of this general survey, participants could provide their e-mail address if they agreed to participate in the subsequent weekly surveys. The introduction statement of the general survey explained the purpose of the study, while emphasizing the discretionary nature of participation, the possibility to withdraw from the study, and the confidential treatment of the data. Hence, written informed consent was offered to all participants. The authors had access to contact details of participants (i.e., email addresses) to send them the questionnaires and to link the repeated measurements of the same individual. Data were fully anonymized prior to the analyses.

#### Measures

We measured positive and negative affect in the weekly surveys with the same nine-item Emmons Mood Indicator scale as in study 1 [36]. Cronbach alpha scores for the positive (*M*(α) = .93, *SD*(α) = .01, range (α) = .92 to .94) and negative affect (*M*(α) = .86, *SD*(α) = .05, range (α) = .80 to .91) scales were satisfactory.

We measured two job demands (workload and cognitive load) and two job resources (autonomy and social support) in the weekly surveys. Workload, cognitive load, and autonomy were each assessed with three items from the Questionnaire on the Experience and Assessment of Work [38]. Social support was assessed with three items from the Job Content Questionnaire [47]. All items were rated on a 4-point Likert scale ranging from 1 (never) to 4 (always). Example items are: “During the past week, I had to work fast” (*workload*); “During the past week, my work required a lot of concentration” (*cognitive load*); “During the past week, I could decide on my own how my work is executed” (*autonomy*); and “During the past week, my colleagues helped me with my tasks” (*social support*). Cronbach alpha scores for the workload (*M*(α) = .81, *SD*(α) = .08, range (α) = .67 to .87), cognitive load (*M*(α) = .85, *SD*(α) = .05, range (α) = .78 to .91), autonomy (*M*(α) = .83, *SD*(α) = .02, range (α) = .80 to .85), and social support (*M*(α) = .76, *SD*(α) = .08, range (α) = .64 to .83) scales were satisfactory. In addition, the cronbach alpha scores of the combined job demands (*M*(α) = .88, *SD*(α) = .04, range (α) = .81 to .90) and job resources (*M*(α) = .75, *SD*(α) = .09, range (α) = .67 to .85) scales were also satisfactory.

We used the same single-item measure to assess PC breach in the weekly surveys as in Study 1 (see S1 Appendix).

We used the general survey to collect demographic information on respondents’ age (in years), gender (female or male), educational background (highest level of formal education), occupational background, average hours volunteered per month, and company tenure (in years). In addition, we measured the extent to which respondents expected job demands and resources. We used the same items that were used to measure actual job demands and resources in the weekly surveys [38,47], but changed the answer format and instructions. The instruction for expected job demands was “Think about your [paid/voluntary] work in [name organization]. Can [name organization] expect from you to…”, while the instruction for expected job resources was “Think about your [paid/voluntary] work in [name organization]. Can you expect from [name organization] to…”. Each item was answered on a 4-item Likert scale ranging from “Totally disagree” (1) to “Totally agree” (4).

### Results and Discussion

#### Confirmatory factor analysis

To test whether job demands and resources, positive and negative affect, and PC breach could empirically be distinguished, we performed a series of multilevel confirmatory factor analyses (CFAs) in Mplus version 7 [41] (detailed description of the analyses can be found in S3 Appendix). Similar to Study 1, we specified the same factor structure at the within- and between-person level in each model. We compared a theory-based model containing seven first-order latent factors (i.e., PC breach, work load cognitive load, autonomy, social support, positive affect, and negative affect) and two second-order latent factors (i.e., job demands [work load and cognitive load] and job resources [autonomy and social support]) to five alternative models (see Table 2). In Alternative model A, the first-order PC breach latent factor loaded on the second-order job demands latent factor. In Alternative model B, the first-order PC breach latent factor loaded on the second-order job resources latent factor. In Alternative model C, the positive and negative affect indicators all loaded on a first-order affect latent factor. Alternative model D contained no second-order latent factors. Alternative model E contained no second-order latent factors, the indicators of cognitive load and work load all loaded directly on a job demands latent factor, and the indicators of autonomy and social support all loaded directly on a job resources latent factor. Alternative models A (Δχ^2^(8) = 39.97, *p* < .001), B (Δχ^2^(8) = 31.13, *p* < .001), C (Δχ^2^(8) = 49.73, *p* < .001), and E (Δχ^2^(3) = 83.15, *p* < .001) fitted significantly worse to the data than the theory-based model. Alternative model D fitted equally well to the data as the theory-based model (Δχ^2^(18) = 11.82, *p* = .86). Given that the Theory-based model and Alternative model D both fitted the data equally well, we conducted two sets of analyses. One in which we focused on job demands and resources in general (see Multilevel path models) and one in which we studied the unique effects of work load, cognitive load, autonomy, and social support (see Sensitivity analyses).

**Table 2:** Results from confirmatory factor analyses in study 2.

Model	*χ*^*2*^ *(df)*	RMSEA	CFI	TLI
Theoretical model	633.63 (420)	.05	.91	.90
Alternative model A	680.81 (428)	.05	.89	.88
Alternative model B	666.92 (428)	.05	.90	.89
Alternative model C	740.79 (428)	.06	.86	.85
Alternative model D	630.83 (402)	.05	.90	.89
Alternative model E	784.97 (423)	.06	.84	.83

*Notes*. *N (within)* = 246, *N (between)* = 100.

*Theoretical model*: *Seven first-order latent factors = PC breach*, *work load*, *cognitive load*, *autonomy*, *social support*, *positive affect*, *negative affect*. *Two second-order latent factors = demands (work load + cognitive load)*, *resources (autonomy + social support)*. *Alternative model A*: *Seven first-order latent factors = PC breach*, *work load*, *cognitive load*, *autonomy*, *social support*, *positive affect*, *negative affect*. *Two second-order latent factors = demands (work load + cognitive load + PC breach)*, *resources (autonomy + social support)*. *Alternative model B*: *Seven first-order latent factors = PC breach*, *work load*, *cognitive load*, *autonomy*, *social support*, *positive affect*, *negative affect*. *Two second-order latent factors = demands (work load + cognitive load)*, *resources (autonomy + social support + PC breach)*. *Alternative model C*: *Six first-order latent factors = PC breach*, *work load*, *cognitive load*, *autonomy*, *social support*, *affect*. *Two second-order latent factors = demands (work load + cognitive load)*, *resources (autonomy + social support)*. *Alternative model D*: *Seven first-order latent factors = PC breach*, *work load*, *cognitive load*, *autonomy*, *social support*, *positive affect*, *negative affect*. *No second-order latent factors*. *Alternative model E*: *Five first-order latent factors = PC breach*, *job demands*, *job resources*, *positive affect*, *negative affect*.

Next, we assessed the presence of common method variance by adding a latent common method factor to the theory-based model. The CMV model with constrained factor loadings fitted the data significantly better than the theory-based model (Δχ^2^(1) = 6.38, *p* = .01). The CMV model with freely estimated factor loadings again improved model fit compared to the CMV model with constrained factor loadings (Δχ^2^(19) = 105.61, *p* < .001). These results suggest that common method variance may be a concern.

#### Multilevel path models

The ICC values of positive mood (.80), negative mood (.63), and PC breach (.41) indicated that a substantive amount of variance was situated at the between-person level. We therefore estimated multilevel path models to test our hypotheses. First, we replicated the results of Study 1 by testing concurrent relationships (i.e., relationships between variables measured at the same point in time). Second, we tested the same model, but with time-lagged relationships to procedurally reduce the influence of common method variance [43] and to examine lingering effects. Time-lagged relationships involve regressing the dependent variable measured at time T on a set of independent variables measured at time T-1, while controlling for the dependent variable at time T-1. By controlling for the dependent variable at the previous point in time, the effect of the previous state of the dependent variable is partialled out, implying that change in the dependent variable is modeled [48]. For similar reasons as in Study 1, we person-mean centered the independent variables prior to testing all models.

**Fig 2:**
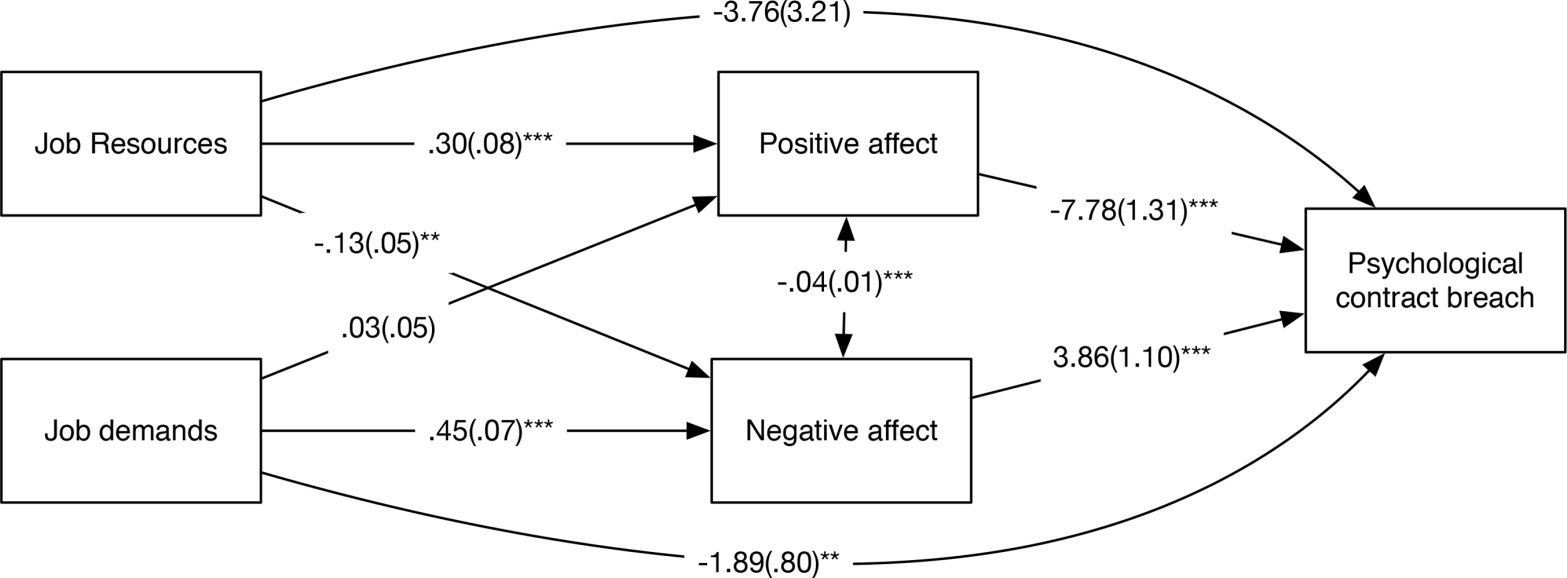
Path estimates from model with concurrent relationships in Study 2. *: *p* < .05, **: *p* < .01, ***: *p* < .001. Standard errors between parentheses.

We first turn to the models with concurrent relationships. Because the partial mediation model was again just identified, we used the BIC values to compare the fit of the partial and the full mediation models. This comparison showed that the full mediation model (χ^2^(2) = 3.04, *p* = .22, RMSEA = .05, CFI = .99, TLI = .96, BIC = 2455.50) offered a better fit to the data than the partial mediation model (χ^2^(0) = 1, *p* < .001, RMSEA = 0, CFI = 1, TLI = 1, BIC = 2465.22). To maximize comparability with the findings from Study 1, we present the results from the partial mediation model (see Fig 2). Hypotheses 1 and 2 could be confirmed as job resources were positively related to positive affect (γ = .30, *p* < .001) and job demands were positively related to negative affect (γ = .45, *p* < .001). Hypotheses 3 and 4 were confirmed as positive affect and negative affect were negatively (β = -7.78, *p* < .001) and positively (β = 3.86, *p* < .001) related to PC breach, respectively. We used the product-of-coefficients approach to estimate indirect effects [44]. Hypothesis 5 could be confirmed as the indirect effect of job resources PC breach, via positive affect (*estimate* = -2.30, *p* < .001) was significant. Hypothesis 6 could be confirmed as the indirect effect of job demands on PC breach, via negative affect (*estimate* = 1.74, *p* = .003) was significant.

**Fig 3:**
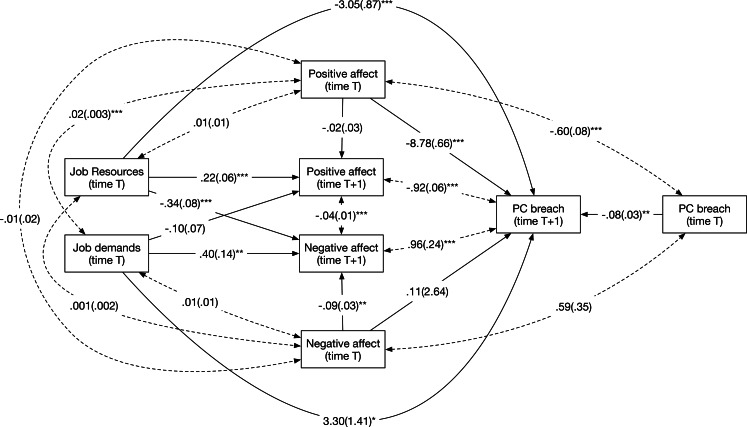
Path estimates from model with time-lagged relationships in Study 2. *: *p* < .05, **: *p* < .01, ***: *p* < .001. Standard errors between parentheses.

Next, we turn to the models with time-lagged relationships. Comparing a full to a partial mediation model revealed that both models fitted equally well to the data (Δχ^2^(2) = 4.79, *p* = .09). We therefore present the results (see Fig 3) from the partial mediation model to maximize comparability with earlier results. The partial mediation model fitted well to the data, except for the RMSEA and TLI indicators (χ^2^(4) = 18.13, RMSEA = .12, CFI = .91, TLI = .35). In addition, the Standardized Root Mean Square Residual was well below the recommended cut-off of .05 at the within- and between-person level (SRMR_within_ = .00, SRMR_between_ = .04), suggesting that the model fit might be satisfactory overall. Hypotheses 1 and 2 could be confirmed as job resources in the present week were positively related to positive affect the subsequent week (γ = .22, *p* < .001) and job demands in the present week were positively related to negative affect the subsequent week (γ = .40, *p* = .01). Hypothesis 3 could also be confirmed as positive affect in the present week was negatively related to PC breach the subsequent week (β = -8.78, *p* < .001). Hypothesis 4 was rejected as negative affect in the present week was not significantly related to PC breach the subsequent week (β = -.11, *p* = .97). Indirect effects were estimated using the products-of-coefficients approach. We found a significant indirect effect of job resources on PC breach via positive emotions (*estimate* = -1.95, *p* < .001), supporting Hypothesis 5. However, we could not find support for an indirect effect of job demands on PC breach via negative affect (*estimate* = .04, *p* = .97), disconfirming Hypothesis 6.

#### Sensitivity analyses

We tested if specific job demands (i.e., work load and cognitive load) and job resources (i.e., autonomy and social support) had similar or distinct effects on perceptions of job breach, via positive and negative affect. We therefore compared a model in which the effects of workload and cognitive load, and of autonomy and social support were constrained to be equal to an unconstrained model in which these effects were freely estimated. The unconstrained model fitted better to the data than the constrained model (Δχ^2^(4) = 43.76, *p* < .001), meaning that specific job demands and resources had unique effects. Regarding the job demands, we found that both workload (γ = .13, *p* = .001) and cognitive load (γ = .25, *p* < .001) in the present week were significantly related to negative affect the subsequent week. With respect to the job resources, social support in the present week was significantly related to positive affect the subsequent week (γ = .36, *p* < .001), while autonomy in the present week was unrelated to positive affect the subsequent week (γ = -.12, *p* = .09).

Next, we assessed if expectations concerning job demands and resources would moderate the relationships between perceived job demands and resources on the one hand and positive and negative affect on the other. To this end, we estimated multilevel moderated mediation models in which expected job demands and resources served as cross-level moderators. This was done using a Bayesian estimator, with 95% credibility intervals (95%CI), because these complex models did not converge with traditional maximum likelihood estimators. The presence of interaction effects is required to support the argument that perceived job demands and resources influence affect only when they exceed the expected level of job demands and resources. The extent to which job demands were expected did not moderate the relationship between job demands and negative affect (β = -.23, 95%CI = [-.73, .32]) or the relationship between job demands and positive affect (β = .33, 95%CI = [-.09, .76]). The extent to which job resources were expected did not moderate the relationship between job resources and negative affect (β = -.11, 95%CI = [-1.73, 1.46]) or the relationship between job resources and positive affect (β = -.01, 95%CI = [-1.05, .92]). Hence, we can conclude that the effect of perceived job demands and resources on the one hand and negative and positive affect on the other hand, did not depend on the extent to which job demands and resources were expected by the respondents.

Finally, we tested if the relationships in our model differed between paid employees and volunteers. We therefore estimated a model in which the relationships between job demands and resources, positive and negative affect, and PC breach were constrained to be equal for paid employees and volunteers. This constrained model was compared to a model in which these relationships were allowed to differ for paid employees and volunteers (i.e., unconstrained model). Results showed that both models fitted equally well to the data (Δχ^2^(24) = 32.23, *p* = .12). Given that the more parsimonious constrained model is to be preferred, we can conclude that volunteers and paid employees react similarly to perceived job demands and resources, in terms of positive and negative affect and PC breach.

When summarizing the findings of our second study, we see that, similar to Study 1, job resources were positively associated with positive affect (*Hypothesis 1*), positive affect was negatively associated with PC breach (*Hypothesis 3*), and positive affect mediated the relationship between job resources and PC breach (*Hypothesis 5*). In contrast to Study 1, job demands were positively associated with negative affect (*Hypothesis 2*), negative affect was positively associated with PC breach (*Hypothesis 4*), and negative affect mediated the relationship between job demands and PC breach (*Hypothesis 6*). We also tested time-lagged relationships, because this allowed us to demonstrate temporal precedence and to procedurally minimize issues related to common method variance [43]. These time-lagged relationships revealed that high job resources and job demands in the present week were associated with high positive and negative affect the subsequent week respectively. In turn, high positive affect in the present week was associated with lower levels of PC breach the subsequent week, and positive affect mediated the relationship between job resources and PC breach. Hypotheses 4 and 6 could not be supported since negative affect in the present week was unrelated to PC breach the subsequent week. A possible explanation for this may be that the influence of negative affect may be short-lived and does not linger for a week. Indeed, the Broaden-and-Build theory of emotions [49] proposes that negative emotions trigger specific and immediate actions, whereas positive emotions build enduring resources that can be used later on. Finally, we performed three sensitivity analyses. These showed that the relationships between job demands and resources on the one hand and positive and negative affect on the other hand, depend on the type of job demand and resource. Some job resources (i.e., social support) and job demands (i.e., cognitive load) were more strongly related to positive and negative affect than others (i.e., autonomy and workload). Second, the relationship between job demands and resources on the one hand and positive and negative affect on the other hand, did not depend on the extent to which demands and resources were expected. This finding aligns with social comparisons theory [17], as self-referent comparisons imply that people compare actual to past job demands and resources as opposed to comparing actual to expected job demands and resources. Third, the relationship between job demands and resources, positive and negative affect, and PC breach, also did not depend on the type of exchange relationship (i.e., volunteers versus paid employees). This suggests that while the content of volunteers’ PCs may differ from that of paid employees, the processes linking antecedents and consequences to PC breach are likely universal.

## General Discussion

We contribute to the PC literature by showing that perceptions of job demands and resources influence perceptions of PC breach and by revealing a possible mediating mechanism underlying this relationship. In particular, we demonstrated that experienced job demands and resources trigger negative and positive affect, which in turn influence individuals’ judgments concerning the extent to which their organization fulfills its obligations. By doing so, we responded to Conway and Briner’s [7] call for more attention to processes relating to the formation of PCs in general and PC breach in particular.

In both studies we found a relationship between job resources and positive affect (i.e., Hypothesis 1). This finding underscores our argument that job resources satisfy basic needs [20] and elicit enduring positive affect [21]. The relationship between job demands and negative affect (i.e., Hypothesis 2) could only be confirmed in Study 2. It appears that job demands do not always hinder need satisfaction and elicit negative affect [20,21]. This result may be due to the fact that we focused on general job demands in Study 1, whereas we focused on workload and cognitive load in Study 2. Alternatively, the difference in findings may be explained by contextual differences between both studies’ samples and organizations. For example, because the first study took place in a youth organization the average age of respondents was lower in Study 1 than in Study 2. It is possible that the younger volunteers in this youth organization interpreted job demands as challenges, rather than as hindrances [50].

Our third Hypothesis—positive affect relates negatively with perceptions of PC breach—was confirmed in Study 1 and Study 2. When employees experience high positive affect, they may primarily recall or pay attention to obligations that were fulfilled by the organization [6]. In other words, positive affect reduces the likelihood that PC breaches are perceived [15]. Hypothesis four—negative affect relates positively to perceptions of PC breach—could only be confirmed in Study 2, and only when testing concurrent relationships. People experiencing high negative affect may focus on negative occurrences when judging if their PC is breached [6]. However, this effect may be short-lived as negative affect may dissipate rapidly, whereas the effects of positive affect may linger because they build enduring resources [49].

Our fifth Hypothesis—positive affect mediates the relationships between job resources and PC breach—was supported in both studies. People who experience high job resources may compare this to their prior experiences and consequently experience positive affect. This in turn buffers against perceiving a breach in their PC [16]. This finding supports recent propositions in PC theory 2.0 [30] that positive affect can increase the likelihood that environmental cues are interpreted as congruent with the PC. It also aligns with the AIM [6]: positive affect increases the likelihood that people will favorably evaluate the fulfillment of their PC because they use positive affect as a heuristic and primarily rely on positive environmental cues. Hypothesis 6 could only be confirmed concurrently in Study 2. The reason might be that, while negative affect may amplify discrepancy detection and, hence, the likelihood to perceive PC breach [30], this effect may be immediate and short-lived [49].

### General limitations

A first limitation of our study is that we did not distinguish challenge from hindrance demands [50]. Challenge demands offer opportunities for personal growth or future gains (e.g. high level of job responsibility) whereas hindrance demands thwart personal growth and future gains (e.g. role conflict). Crawford and colleagues [50] argue that aggregating both types of demands may result in weaker or statistically non-significant relationships between generalized demands and outcomes.

Second, we argued that experienced job demands and resources either thwart or satisfy basic needs [20], which in turn leads to changes in positive and negative affect [21]. However, we did not include basic need satisfaction as a mediator in the relationship between job demands and resources and affect. Including basic need satisfaction may help unravel why some job demands and resources (do not) trigger changes in affect in samples of paid employees and volunteers. Likewise, we based our arguments on Social Comparison Theory [17], arguing that people engage in self-referent comparisons and compare their current levels of perceived job demands and resources to typical, past levels of perceived job demands and resources. We captured these self-referent comparisons indirectly by person-mean centering the data. However, a more explicit, direct measure of self-referent comparisons may help to further model and understand this process. For example, explicitly asking respondents to rate current job demands and resources while comparing them to their own past experiences or to the experiences of their co-workers, could help to disentangle the effects of self-referent and other-referent comparisons.

Third, we used a new single-item measure of PC breach in both studies. We developed this new measure because traditional measures often fail to refer to a specific period in time. Hence, these traditional scales are not ideal in experience sampling research. Moreover, we wanted to treat PC breach as a continuum ranging from under-fulfilment of obligations, over exact fulfilment of obligations, to over-fulfilment of obligations. Traditional measures typically only consider under-fulfilment as PC breach, and as a result these measures may not capture the entire continuum [35]. Nonetheless, we acknowledge that this single-item measure has certain limitations. First, it is a general measure of breach—similar to many traditional measures—which does not refer to specific elements in the PC. General PC breach measures may be less biased than composite PC breach measures because they do not a priori define the content of the PC and because they allow respondents to subjectively weigh the importance and salience of all elements in their PC [3]. Nevertheless, it might be interesting for future research to look into the differential roles of antecedents of transactional, relational, and ideological PC breach. Second, our single-item measure anthropomorphizes the organization, meaning that it does not distinguish between different actors (e.g., supervisor, HR-staff) that may have caused the PC breach. Recent research has shown that respondents indeed consider multiple actors to jointly represent the organization when reflecting on PC breach [12]. Future studies could examine whether reactions to PC breach differ depending on the organizational actor associated with the breach [51]. Despite these limitations, we believe that our measure was suited for studying PC breach in experience sampling research; a belief that was further supported by the measure’s successful cross-validation.

Finally, our samples in studies 1 and 2 may not be representative for the entire population of volunteers and paid employees. While the response rates in both studies may appear low at first sight, they aligned with those typically found in similar research [e.g. 11]. Nonetheless, a happy-worker bias may be present, as unhappy volunteers and employees—possibly unhappy due to a PC breach—may have self-selected out of the survey.

### General implications for research and practice

Our findings reveal a number of interesting avenues for future research. First, the role of affect as an antecedent of PC breach should be explored further. While a number of other antecedents such as trust [8] and Human Resource practices [10] have been identified, it is unknown how these antecedents lead to perceptions of PC breach. Introducing affect as a mediator could help to elucidate these relationships. Second, given that affect plays a role in the emergence of PC breach perceptions, it appears worthwhile to investigate potential moderators such as emotion-regulation strategies [52]. This may help determine the conditions under which affect is most likely to infuse PC breach judgments. Third, we believe that more attention could be paid to studying affect as an outcome of PC breach. The PC literature focuses on feelings of violation as an emotional response to PC breach [15], yet little is known with regard to the affective reactions or specific discrete emotions underlying violation. Post-hoc analyses in Study 2 indeed confirmed that perceptions of breach in the present week were positively related to negative affect (γ = .25, *p* < .001) and negatively related to positive affect the subsequent week (γ = -.14, *p* = .04). Moreover, these relationships were the same for volunteers and for paid employees (χ^2^(2) = 5.58, *p* = .06). Hence, affect may simultaneously act as an antecedent and a consequent of PC breach. Lastly, one should take into account that there is ample variation within the volunteer population. For example, it has been argued that the PC of volunteers may depend on their volunteering style [53]. For example, while some job demands such as high workload may be acceptable for individuals with a traditional volunteering style, they may be undesirable for individuals with an episodic volunteering style. Future studies could explore if these differences in volunteering style explain why some job demands and resources are more relevant to some volunteers than to others.

A first important implication for practice is that suggestions to manage PC breach of paid employees may equally apply to volunteers, as our findings suggest that the processes leading to PC breach perceptions are the same for both groups. Second, it is important to consider that perceptions of job demands and resources, positive and negative affect, and PC breach vary over time. Organizations should therefore not only focus on crafting jobs low on demands and high on resources, but also monitor closely when periods with high demands and/or low resources occur. During these periods, attention should be paid to regulating volunteers’ and paid employees’ emotions to prevent PC breach. For example, organizations could provide opportunities for volunteers and employees to discuss and vent their emotions with supervisors; organize events that elicit positive affect (e.g. social gatherings); or cognitively reappraise perceptions of demands and resources [52]. In addition, selecting supervisors with strong emotional intelligence could help manage volunteers’ and paid employees’ emotional reactions [54]. Supervisors should react swiftly to prevent negative affect from triggering PC breach perceptions, while investing in job resources to stimulate positive affect may be a successful long-term strategy to build enduring personal resources that prevent PC breach perceptions from arising.

## Conclusion

Based on two experience sampling studies, we set out to gain a better understanding of experienced job demands and resources as antecedents of PC breach. Our findings confirm that when employees perceive high demands and/or low resources they are more likely to report that their organization is not fulfilling its obligations. Moreover, we show that this relationship can be explained by positive and negative affect, albeit the latter only in the short-term.

## Supporting Information

S1 Appendix Psychological contract breach item. (DOCX)

S2 Appendix Cross-validation of measures. (DOCX)

S3 Appendix Detailed description of analyses. (DOCX)
