# Supplementary material for: Antecedents of Psychological Contract Breach: The Role of Job Demands, Job Resources, and Affect
Source: PLoS One. 2016 May 12;11(5):e0154696. doi: 10.1371/journal.pone.0154696 (PMC4865204; doi:10.1371/journal.pone.0154696)
Supplement: S1 Appendix — (DOCX) [file pone.0154696.s001.docx]

**Appendix 1: Psychological contract breach item**

Far less fulfilled than expected

Far more fulfilled than expected

Exactly fulfilled as expected

Figure 1. Psychological contract breach item used in Study 1 and Study 2.

Instruction for item: “Everyone perceives that their organization has certain obligations towards them, but these obligations are not always fulfilled. Please indicate on the following line to what extent your organization fulfilled its obligations towards you during the past day (Study 1) / week (Study 2).”
