# Supplementary material for: Antecedents of Psychological Contract Breach: The Role of Job Demands, Job Resources, and Affect
Source: PLoS One. 2016 May 12;11(5):e0154696. doi: 10.1371/journal.pone.0154696 (PMC4865204; doi:10.1371/journal.pone.0154696)
Supplement: S2 Appendix — (DOCX) [file pone.0154696.s002.docx]

**Appendix 2: Cross-validation of measures**

**Psychological contract breach**

We cross-validated the single-item measure of psychological contract breach by correlating it with a traditional breach measure by Tekleab and Taylor [1]. Both measures were included in the weekly surveys in Study 2. Tekleab and Taylor’s scale consists of three items (e.g., “(Name organization) has repeatedly failed to meet its obligations to me during the past week”). We added “during the past week” to all items in Tekleab and Taylor’s scale, to make sure that all items referred to the appropriate timeframe. Responses on these items were given on a five-point Likert scale ranging from “Totally disagree” (1) to “Totally agree” (5).

To demonstrate convergent validity, we calculated the correlation between both measures. As the data are nested (5 weekly observations per respondent), we estimated both within- and between-person correlations in a multilevel model in Mplus version 7. Results showed that there was a statistically significant positive correlation between the single-item measure and Tekleab and Taylor’s measure of breach at the within- (*r*=.38, *p*<.001) and at the between-person (*r*=.38, *p*=.01) level. Hence, these correlations confirm the convergent validity of the single-item measure of psychological contract breach.

**Job demands**

We cross-validated the single-item job demands measure from Study 1 by correlating it with a job demands scale from the Questionnaire on the Experience and Assessment of Work by van Veldhoven and Meijman [2]. Both measures were included in the general survey in Study 2. The measure by van Veldhoven and Meijman contained 6 items measuring workload and cognitive load (e.g., “My work requires a lot of concentration”). These items were rated on a 4-point Likert scale ranging from 1 (never) to 4 (always). As both measures were included in the general survey of Study 2, there was no nested structure and we could use a Pearson correlation coefficient to demonstrate convergent validity. Results showed that the single-item job demands measure correlated significantly and positively with the six-item job demands measure by van Veldhoven and Meijman (*r*=.57, *p*<.001). This finding supports the convergent validity of the single-item job demands measure.

**Job resources**

We cross-validated the two-item job resources measure from Study 1 by correlating it with a job resources scale based on the Questionnaire on the Experience and Assessment of Work by van Veldhoven and Meijman [2] and the Job Content Questionnaire by Karasek and colleagues [3]. Both measures were included in the general survey in Study 2. We used three items from the scale by van Veldhoven and Meijman measuring autonomy (e.g., “I can decide on my own how my work is executed”) and three items from the scale by Karasek and colleagues [3] measuring social support (e.g., “My colleagues help me with my tasks”). These items were rated on a 4-point Likert scale ranging from 1 (never) to 4 (always). As both measures were included in the general survey of Study 2, there was no nested structure and we could use a Pearson correlation coefficient to demonstrate convergent validity. Results showed that the two-item job resources measure correlated significantly and positively with the six-item job resources measure (*r*=.45, *p*<.001). This finding supports the convergent validity of the two-item job resources measure.

**References**

1. Tekleab AG, Taylor MS (2003) Aren’t there two parties in an employment relationship? Antecedents and consequences of organization-employee agreement on contract obligations and violations. J Organ Behav 24: 585–608. doi:10.1002/job.204.
2. van Veldhoven MJPM, Meijman T (1994) Het meten van psychosociale arbeidsbelasting met een vragenlijst: De vragenlijst beleving en beoordeling van de arbeid (VBBA).
3. Karasek R, Brisson C, Kawakami N, Houtman I, Bongers P, et al. (1998) The Job Content Questionnaire (JCQ): an instrument for internationally comparative assessments of psychosocial job characteristics. J Occup Health Psychol 3: 322–355. doi: 10.1037/1076-8998.3.4.322
