# Supplementary material for: Antecedents of Psychological Contract Breach: The Role of Job Demands, Job Resources, and Affect
Source: PLoS One. 2016 May 12;11(5):e0154696. doi: 10.1371/journal.pone.0154696 (PMC4865204; doi:10.1371/journal.pone.0154696)
Supplement: S3 Appendix — (DOCX) [file pone.0154696.s003.docx]

**Appendix 3: Detailed description of analyses**

**Study 1**

***Confirmatory factor analysis***

We used confirmatory factor analysis (CFA) in Mplus version 7 to test the factor structure of our measures. A multilevel approach was used to estimate these CFA models, as we had nested data: daily observations were nested within respondents. We hence have two levels of analysis: a within-person level and a between-person level. Multilevel analysis partitions the total variance in a within- and a between- component. Consequently, multilevel CFA allows for testing the factor structure of the measures on the within- and between-person level simultaneously. Models were estimated using the robust maximum likelihood estimator (MLR).

We started by estimating a multilevel CFA model that matched the theoretical factor structure. This means that the items used to measure job demands (i1), job resources (i2-i3), positive affect (i4-i7), negative affect (i8-i12), and PC breach (i13) had factor loadings to their respective latent factors (see Figure 1, top left panel). The double-arrowed lines in Figure 1 indicate that the latent factors were allowed to co-vary with each other at both levels. The singles-arrow grey lines represent measurement error. The factor structure was mirrored at the within- and between-person level, as we did not hypothesize any differences between both levels. We assessed if this theory-based model fitted the data by examining three indices: the Comparative Fit Index (CFI), the Tucker-Lewis Index (TLI), and the Root Mean Square Error of Approximation (RMSEA). The CFI and TLI are goodness-of-fit indices, meaning that high values indicate good model fit. CFI and TLI values above .90 can be interpreted as adequate and values above .95 as good model fit [1]. The RMSEA is a badness-of-fit index, meaning that low values indicate good model fit. Values below .08 can be interpreted as adequate model fit and values below .05 as good model fit [1]. We also report the χ^2^-value and it’s *p*-value, as this is commonly done in CFA models. A *p*-value below .05 indicates good model fit. However, following recommendation in the literature [2], we do not use the χ^2^-value and it’s *p*-value to evaluate model fit, as these depend strongly on sample size. Based on these indices, we can conclude that the theory-based model offered an adequate fit to the data (χ^2^(128)=263.34, *p*<.001; CFI=.93; TLI=.91; RMSEA=.06). All standardized factor loadings were statistically significant and larger than .40 [3].

Next, we compared this theory-based CFA model to three alternative models to rule out alternative factor structures and to further establish the construct validity of our measures. In the first alternative model (Alternative A – see Figure 1, top right panel), the latent factor representing PC breach was removed and the PC breach item (i13) was allowed to load on the latent job demands factor. The alternative A CFA model fitted adequately to the data (χ^2^(134)=262.95, *p*<.001; CFI=.93; TLI=.92; RMSEA=.06). A χ^2^-difference test showed that the theory-based CFA model and the alternative A CFA model fitted equally well to the data (χ^2^(6)=2.26, *p*=.89). However, the standardized factor loading of the job demands item (i1) was smaller than .40 and was not statistically significant (λ=-.09, *p*=.24). We therefore rejected the alternative A CFA model in favor of the theory-based CFA model. In the second alternative model (Alternative B – see Figure 1, bottom left panel), the latent factor representing PC breach was removed and the PC breach item (i13) was allowed to load on the latent job resources factor. The alternative B CFA model did not offer a good fit to the data (χ^2^(136)=485.28, *p*<.001; CFI=.81; TLI=.78; RMSEA=.09). A χ^2^-difference test (corrected for the use of a robust maximum likelihood estimator) showed that the theory-based CFA model fitted significantly better to the data than the alternative B CFA model (χ^2^(8)=162.57, *p*<.001). We therefore rejected the alternative B CFA model in favor of the theory-based CFA model. In the third alternative model (alterative C – see Figure 1, bottom right panel), the latent factors representing positive affect and negative affect were replaced by a general affect latent factor. The positive affect (i4-i7) and the negative affect (i8-i12) items were all allowed to load on this general affect latent factor. The alternative C CFA model did not offer a good fit to the data (χ^2^(137)=578.29, *p*<.001; CFI=.76; TLI=.73; RMSEA=.11). A χ^2^-difference test showed that the theory-based CFA model fitted significantly better to the data than the alternative C CFA model (χ^2^(9)=151.00, *p*<.001). We therefore rejected the alternative C CFA model in favor of the theory-based CFA model.

We examined if common method bias was present in our data, because we used self-reported surveys to measure all variables [4]. We therefore re-estimated the abovementioned theory-based CFA model, but included a latent common method factor (see Figure 2). This latent common method factor was introduced only at the within-person level, as all our hypotheses pertain to this level. All items had factor loadings on the latent common method factor (dashed single-arrow lines in Figure 2), next to the factor loadings to their own latent factors. In a first CFA model, all the factor loadings of the latent common method factor were constrained to be equal to each other (constrained CMV model). This represents the case where common method has an equal influence on respondents’ answers to all survey items. A χ^2^-difference test showed that the constrained CMV model did not improve model fit, compared to the theory-based model (χ^2^(2)=3.80, *p*=.15). In a second CFA model, all the factor loadings of the latent common method factor were freely estimated (unconstrained CMV model). This represents the case where common method differentially influences respondents’ answers to all survey items. A χ^2^-difference test showed that the unconstrained CMV model did not improve model fit, compared to the theory-based model (χ^2^(13)=21.77, *p*=.06). We therefore concluded that common method variance did not influence respondents’ responses to survey items.

******

*Figure 1. Overview of CFA models estimated in Study 1.*

***
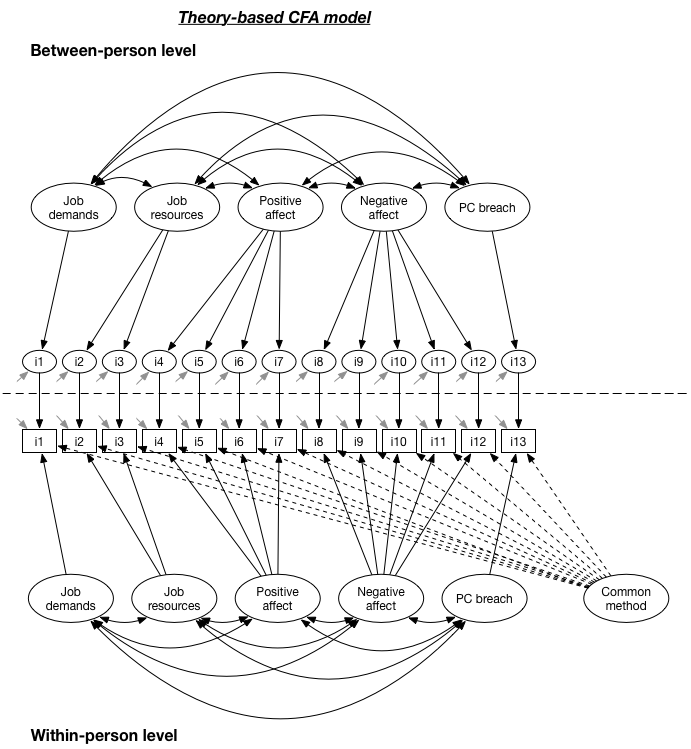
***

*Figure 2. CFA model with latent common method factor in Study 1.*

***Multilevel path model***

We estimated multilevel path models to test our hypotheses. Two models were estimated: a full mediation model (see Figure 3) and a partial mediation model (see Figure 4). These models contained two exogeneous variables (i.e., variables that are not caused by other variables in the model), namely job demands and job resources, and three endogeneous variables (i.e., variables that are caused by other variables), namely positive affect, negative affect, and PC breach. Paths from exogeneous to endogeneous variables are represented by γ, while paths between endogeneous variables are represented by β. A covariance between variables is represented by φ. We person-mean centered all independent variables, meaning that we subtracted the average of a variable for a person from the daily scores of that person on the same variable. Consequently, we removed all between-person variance from the variables, as we were only interested in within-person relationships. Hypotheses were assessed by examining the path estimates at the within-person level.

We calculated Intraclass Correlation Coefficients (ICC) of the endogeneous variables prior to testing hypotheses. ICC values represent the ratio of between-person variance to the total variance (within-person + between person):

$$ICC= \frac{\sigma_{between}^{2}}{\sigma_{between}^{2}+\sigma_{within}^{2}}$$

If an ICC value is larger than .10, there is a substantive amount of variance at the between-person level meaning that multilevel analyses are required [5]. The ICC values of positive affect (ICC=.16), negative affect (ICC=.27), and PC breach (ICC=.15) indicated that a multilevel approach was necessary.

We started by comparing a full (i.e., the relationship between job demands/resources and PC breach is fully accounted for by positive and negative affect) to a partial mediation model (i.e., the relationship between job demands/resources and PC breach is partially accounted for by positive and negative affect). Because the partial mediation model was just-identified (i.e., no degrees of freedom left), we compared models by looking at the Bayesian Information Criterion (BIC). Smaller BIC values indicate better model fit. This comparison revealed that the partial mediation model fitted the data (*χ^2^*(0)=0 , RMSEA=0, CFI=1, TLI=1, BIC=3543.23) better than the full mediation model (*χ^2^*(2)=10.68, RMSEA=.12, CFI=.95, TLI=.76, BIC=3543.92).

At the within-person level, the significant positive relationship between job resources and positive affect (γ=.44, *p*<.001) indicates that volunteers reported higher levels of positive affect on days when they also perceived high job resources. This finding offers support for Hypothesis 1. There was no significant relationship between job demands and negative affect (γ=.06, *p*=.12), disconfirming Hypothesis 2. However, negative affect was significantly related to job resources (γ=-.12, *p*=.01), meaning that volunteers reported lower levels of negative affect on days that they perceived a high amount of job resources. There was a significant negative relationship between PC breach and positive affect (β=-9.37, *p*<.001), but not between PC breach and negative affect (β=2.11, *p*=.27). This offered support for Hypothesis 3, but disconfirmed Hypothesis 4. Put differently, volunteers reported lower levels of PC breach on days that they experienced high levels of positive affect. Job demands (γ=1.75, *p*=.04) and job resources (γ=-3.58, *p*=.002) also had direct effects on perceptions of PC breach.


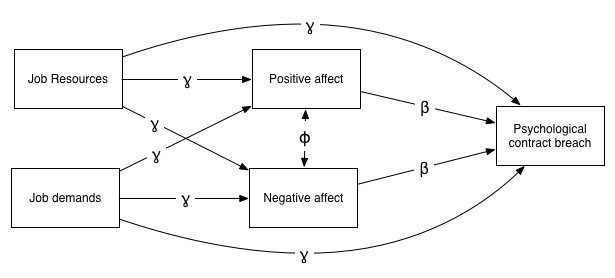


*Figure 3. Full mediation model of Study 1.*


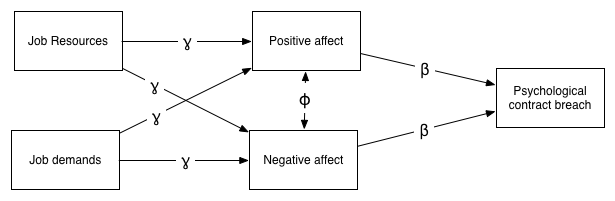


*Figure 4. Partial mediation model of Study 4.*

*Figure 5. Path estimates of partial mediation model.*

To test the mediation effect proposed in Hypothesis 5, we estimated indirect effects using the product-of-coefficients approach. According to this approach, the point estimate of the indirect effect is the product of the path estimate from the independent variable to the mediator, and the path estimate from the mediator to the dependent variable ($\hat{a}\hat{b}$). For example, the indirect effect of job resources on PC breach, via positive affect can be obtained by multiplying .44 ($\hat{a}$ path) and -9.49 ($\hat{b}$ path). The standard error of this product can be obtained using the following formula and the delta method:

$$SE=\sqrt{\hat{a}^{2}s_{b}^{2}+\hat{b}^{2}s_{a}^{2}+s_{a}^{2}s_{b}^{2}}$$

This standard error (*SE*) can be used in a *z*-test to determine the statistical significance of $\hat{a}\hat{b}$. Table 1 displays the point estimates for all indirect effects, their standard errors, and *p*-values. Two of these four indirect effects were used to test hypotheses 5 and 6. There was a significant indirect effect of job resources on psychological contract breach via positive affect (*Point* *estimate*=4.16, *p*<.001). In other words, on days that volunteers perceived high levels of job resources they reported low levels of psychological contract breach, because they experienced high positive affect that day. The indirect effect of job demands on psychological contract breach via negative affect was not significant (*Point* *estimate*=-.13, *p*=.40). Hence, these findings support Hypothesis 5, but disconfirm Hypothesis 6.

*Table 1. Indirect effects in Study 1.*

| Indirect effect | Point estimate | *SE* | *p* | Conclusion |
| --- | --- | --- | --- | --- |
| Job resources 🡪 Positive affect 🡪 PC breach | -4.08 | .83 | .000 | Hypothesis 5 supported |
| Job resources 🡪 Negative affect 🡪 PC breach | -.26 | .23 | .26 | Not used to test hypotheses |
| Job demands 🡪 Positive affect 🡪 PC breach | .99 | .61 | .10 | Not used to test hypotheses |
| Job demands 🡪 Negative affect 🡪 PC breach | .12 | .14 | .40 | Hypothesis 6 rejected |

**Study 2**

***Confirmatory factor analysis***

We used confirmatory factor analysis (CFA) in Mplus version 7 to test the factor structure of our measures. A multilevel approach was used to estimate these CFA models, given that we had nested data: weekly observations were nested within respondents. Models were estimated using the robust maximum likelihood estimator (MLR).

We started by estimating a multilevel CFA model that matched the theoretical factor structure. This means that the items used to measure cognitive load (i1-i3), workload (i4-i6), social support (i7-i9), autonomy (i10-i12), positive affect (i13-i16), negative affect (i17-i21), and PC breach (i22) loaded on their respective latent factors (see Figure 6, top panel). In addition, the latent factors for cognitive load and workload loaded on a latent job demands factor and the latent factors for social support and autonomy loaded on a latent job resources factor. These latent job demands and job resources factors are second-order latent factors, whereas the other latent factors in the model are first-order latent factors. The factor structure was mirrored at the within- and between-person level as we did not hypothesize any differences between both levels. Inspection of the model fit indices of the theory-based model showed that it offered an adequate to good fit to the data (χ^2^(420)=633.63, *p*<.001; CFI=.91; TLI=.90; RMSEA=.05).

Next, we compared this theory-based CFA model to five alternative models to rule out alternative factor structures and to further establish the construct validity of our measures. In the first alternative model (Alternative A – see Figure 6, bottom panel), the latent factor representing PC breach was removed and the PC breach item (i22) was allowed to load on the second-order latent job demands factor. The alternative A CFA model did not fit adequately to the data (χ^2^(428)=680.81, *p*<.001; CFI=.89; TLI=.88; RMSEA=.05). A χ^2^-difference test showed that the theory-based CFA model fitted significantly better to the data than the alternative A CFA model (χ^2^(8)=39.97, *p*<.001). We therefore rejected the alternative A CFA model in favor of the theory-based CFA model. In the second alternative model (Alternative B – see Figure 7, top panel), the latent factor representing PC breach was removed and the PC breach item (i22) was allowed to load on the second-order latent job resources factor. The alternative B CFA model offered a bad to adequate fit to the data (χ^2^(428)=666.92, *p*<.001; CFI=.90; TLI=.89; RMSEA=.05). A χ^2^-difference test showed that the theory-based CFA model fitted significantly better to the data than the alternative B CFA model (χ^2^(8)=31.13, *p*<.001). We therefore rejected the alternative B CFA model in favor of the theory-based CFA model. In the third alternative model (alternative C – see Figure 7, bottom panel), the latent factors representing positive affect and negative affect were replaced by a general affect latent factor. The positive affect (i13-i16) and the negative affect (i17-i21) items were all allowed to load on this general affect latent factor. The alternative C CFA model did not offer a good fit to the data (χ^2^(428)=740.79, *p*<.001; CFI=.86; TLI=.85; RMSEA=.06). A χ^2^-difference test showed that the theory-based CFA model fitted significantly better to the data than the alternative C CFA model (χ^2^(8)=49.73, *p*<.001). We therefore rejected the alternative C CFA model in favor of the theory-based CFA model. In the fourth alternative model (alternative D – see Figure 8, top panel), the second-order latent factors representing job demands and job resources were removed. The alternative D CFA model fitted bad to adequately to the data (χ^2^(402)=630.83, *p*<.001; CFI=.90; TLI=.89; RMSEA=.05). A χ^2^-difference test showed that the theory-based CFA model fitted equally well to the data as the alternative D CFA model (χ^2^(18)=11.82, *p*=.86). We will therefore pay attention to general job demands and job resources (see main path models of Study 2) and to specific job demands (cognitive load and workload) and job resources (social support and autonomy) (see sensitivity analysis of Study 2). In the fifth alternative model (Alternative E – see Figure 8, bottom panel), the first-order latent factors representing cognitive load, workload, social support, and autonomy were removed. The items measuring cognitive load (i1-i3) and workload (i4-i6) loaded directly onto a general job demands latent factor, while the items measuring social support (i7-i9), and autonomy (i10-i12) loaded directly on a general job resources latent factor. The alternative E CFA model offered a bad fit to the data (χ^2^(423)=784.07, *p*<.001; CFI=.84; TLI=.83; RMSEA=.06). A χ^2^-difference test showed that the theory-based CFA model fitted significantly better to the data than the alternative E CFA model (χ^2^(3)=83.15, *p*<.001).

Because we used self-reported surveys to measure all variables we once more tested if common method bias was present in our data [4]. We therefore re-estimated the abovementioned theory-based CFA model, but included a latent common method factor (see Figure 9). This latent common method factor was introduced only at the within-person level as all our hypotheses pertain to this level. All items had factor loadings on the latent common method factor (dashed single-arrow lines in Figure 9), next to the factor loadings to their own latent factors. In a first CFA model, all the factor loadings of the latent common method factor were constrained to be equal to each other (constrained CMV model). This represents the case where common method has an equal influence on respondents’ answers to all survey items. A χ^2^-difference test showed that the constrained CMV model improved model fit, compared to the theory-based model (χ^2^(1)=6.38, *p*=.01). In a second CFA model, all the factor loadings of the latent common method factor were freely estimated (unconstrained CMV model). This represents the case where common method differentially influences respondents’ answers to all survey items. A χ^2^-difference test showed that the unconstrained CMV model improved model fit, compared to the theory-based model (χ^2^(19)=105.61, *p*<.001). We therefore concluded that common method variance may have influenced respondents’ responses to survey items. We will therefore estimate a model with time-lagged relationships—next to a model with momentary relationships—to separate independent and dependent variables in time. This approach procedurally minimizes the impact of common method variance [4].

***
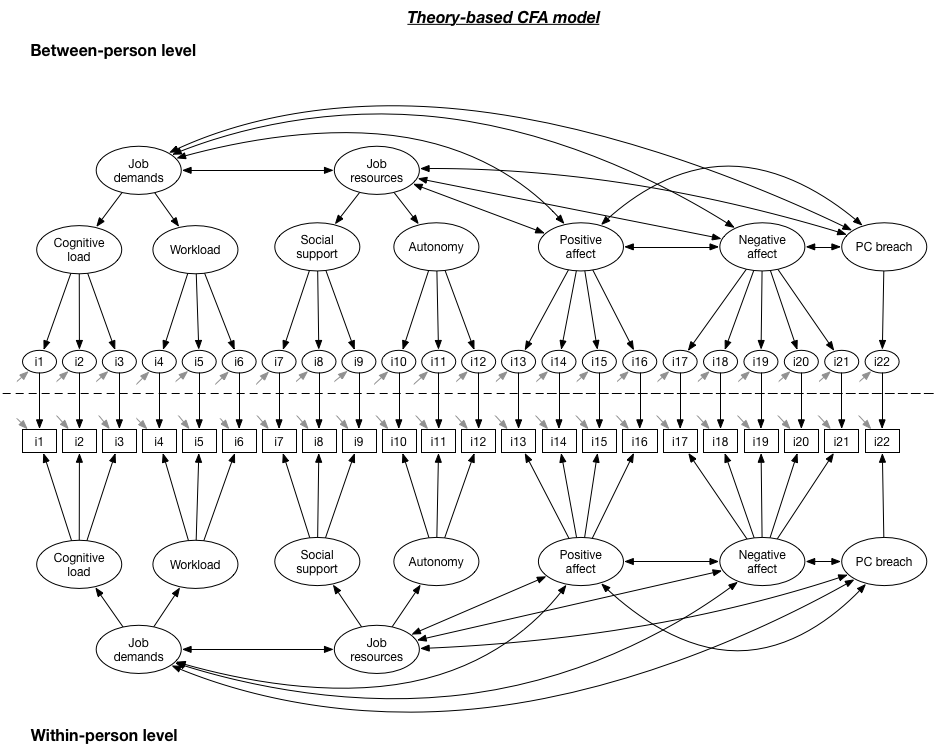
***

***
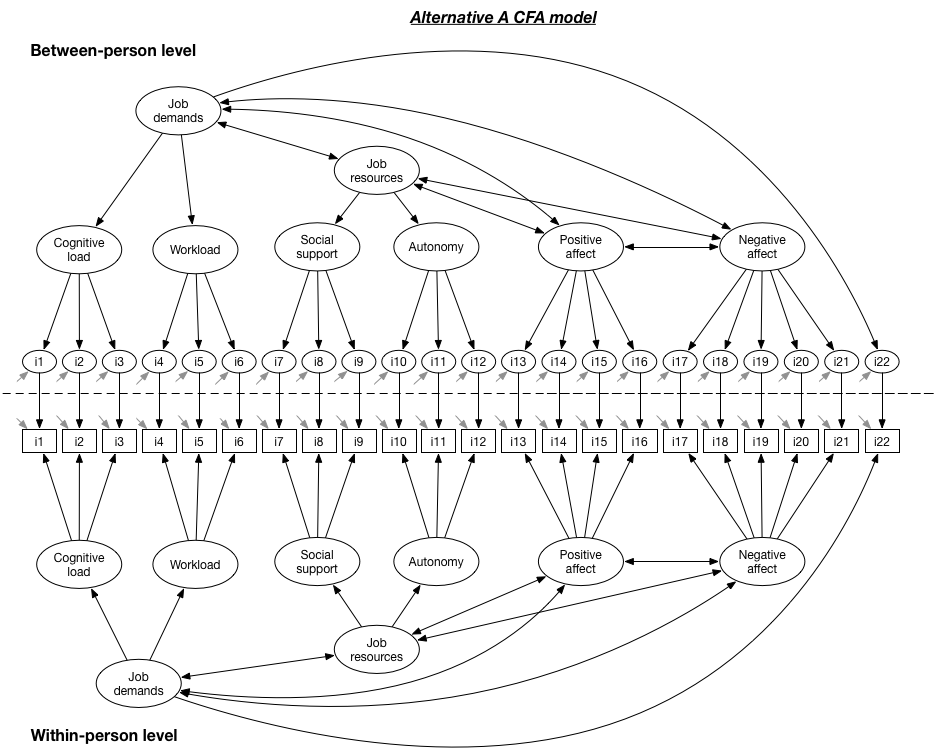
***

*Figure 6. Theory-based and Alternative A CFA models from Study 2.*

***
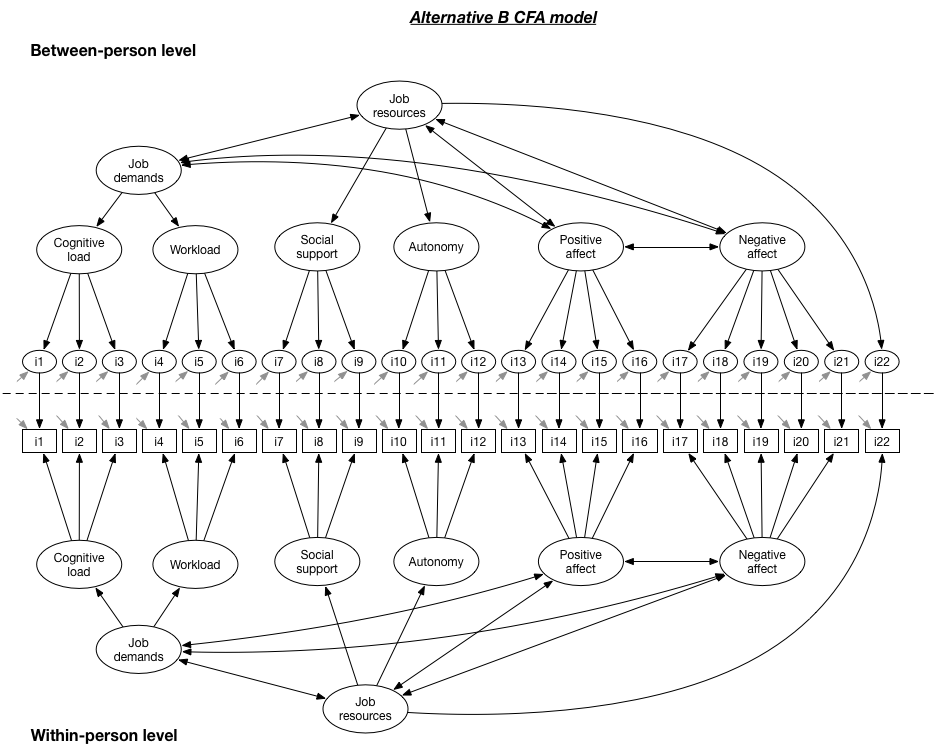
***

***
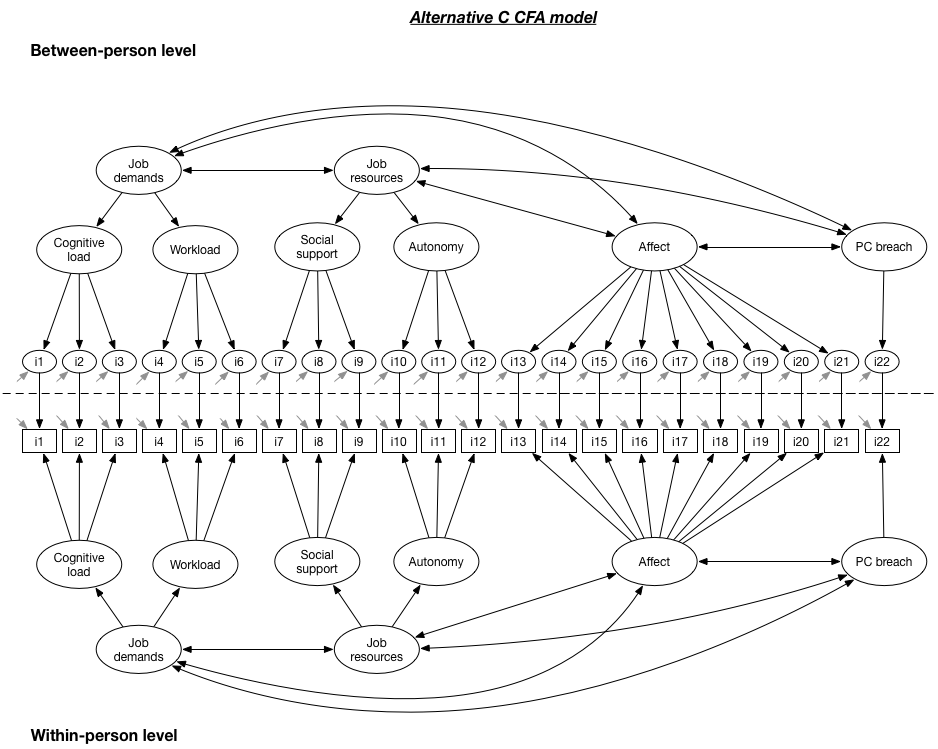
***

*Figure 7. Alternative B and Alternative C CFA models from Study 2.*

***
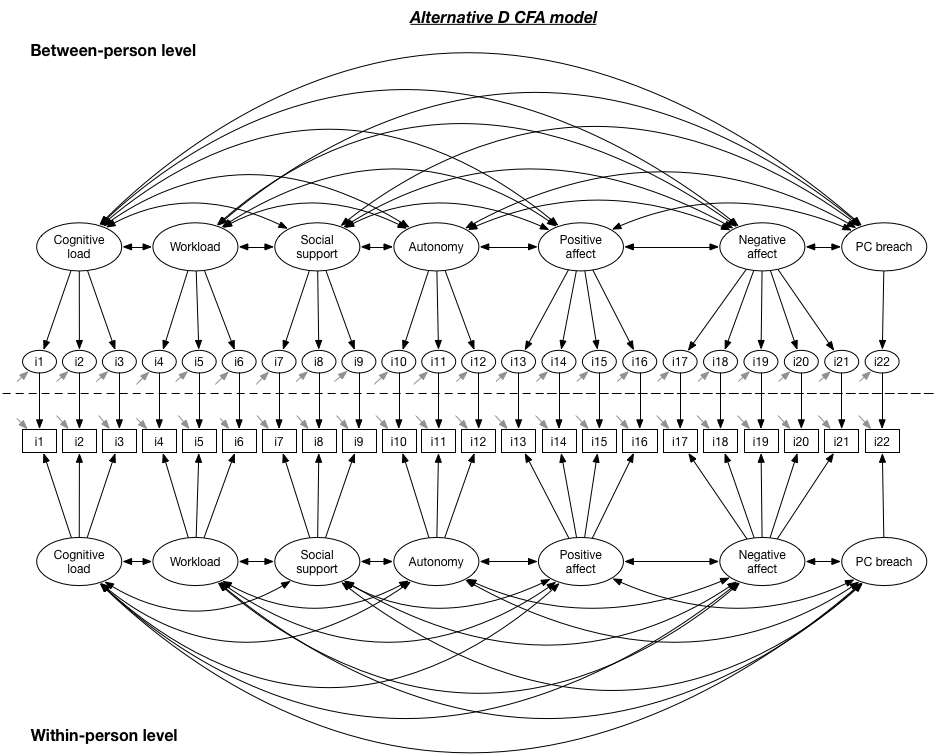
***

***
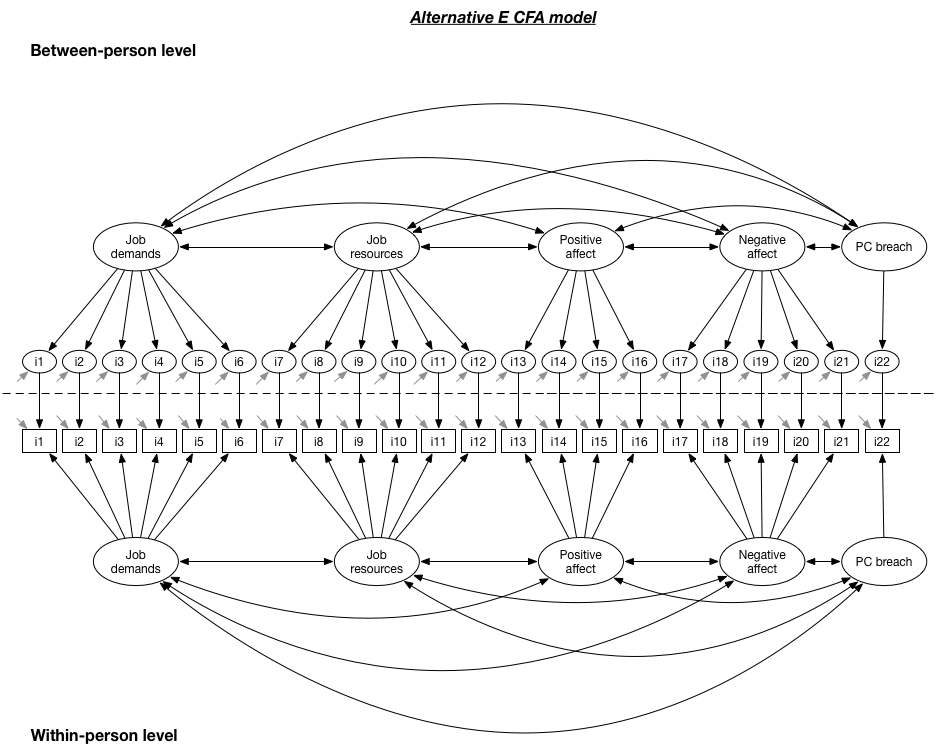
***

*Figure 8. Alternative D and Alternative E CFA models from Study 2.*

***
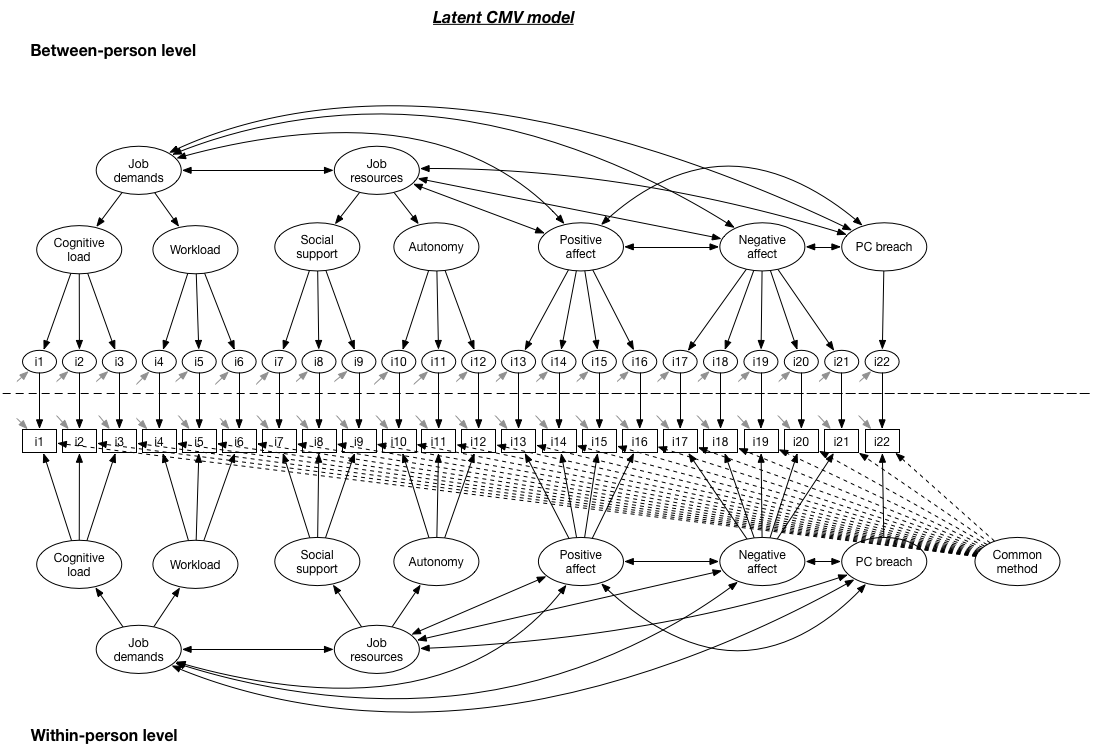
***

*Figure 9. Latent common method variance CFA model from Study 2.*

***Multilevel path models***

**Momentary relationships.** We used multilevel path models to test our hypotheses. We started by estimating two models with momentary relationships (i.e., relationships between variables measured at the same point in time): a full mediation model (see Figure 3) and a partial mediation model (see Figure 4). The reason for estimating these models with momentary relationships is that it allowed us to assess the replicability of the findings from Study 1. We calculated Intraclass Correlation Coefficients (ICC) of the endogeneous variables prior to testing hypotheses. The ICC values of positive affect (ICC=.80), negative affect (ICC=.63), and PC breach (ICC=.41) indicated that using a multilevel approach was necessary. For similar reasons as in Study 1, we person-mean centered the independent variables prior to testing all models.

Because the partial mediation model was again just identified, we used the BIC values to compare the fit of the partial and the full mediation models. This comparison showed that the full mediation model (χ^2^(2)=3.04, *p*=.22, RMSEA=.05, CFI=.99, TLI=.96, BIC=2455.50) offered a better fit to the data than the partial mediation model (χ^2^(0)=1, *p*<.001, RMSEA=0, CFI=1, TLI=1, BIC=2465.22). To maximize comparability with the findings from Study 1, we present the results from the partial mediation model. At the within-person level, the significant positive relationship between job resources and positive affect (γ=.30, *p*<.001) indicates that respondents reported higher levels of positive affect on days that they also perceived high job resources. This finding offers support for Hypothesis 1. The significant positive relationship between job demands and negative affect (γ=.45, *p*<.001), indicates that respondents reported higher levels of negative affect on days that they also perceived high job demands, thus confirming Hypothesis 2. Negative affect was also significantly related with job resources (γ=-.13, *p*=.009), meaning that volunteers reported lower levels of negative affect on days that they perceived a high amount of job resources. There was a significant relationship between PC breach and positive affect (β=-7.78, *p*<.001), and between PC breach and negative affect (β=3.86, *p*<.001). In other words, on days that respondents experienced high positive affect, they also reported lower levels of PC breach; on days that respondents experienced high negative affect, they also reported higher levels of PC breach. This offered support for Hypotheses 3 and 4. Job demands (γ=-1.89, *p*=.02) also had a direct effect on perceptions of PC breach, while job resources was not directly related to PC breach (γ=-3.76, *p*=.24).

We used the product-of-coefficients approach to estimate indirect effects. Hypothesis 5 could be confirmed as the indirect effects of job resources on PC breach via positive affect (*Point* *estimate*=-2.30, *p*<.001) was significant. Hypothesis 6 could be supported as the indirect effect of job demands on PC breach via negative affect (*Point* *estimate*=1.74, *p*=.003) was significant.

*Table 2. Indirect effects in Study 2 – momentary relationships.*

| Indirect effect | Point estimate | *SE* | *p* | Conclusion |
| --- | --- | --- | --- | --- |
| Job resources 🡪 Positive affect 🡪 PC breach | -2.30 | .57 | .000 | Hypothesis 5 supported |
| Job resources 🡪 Negative affect 🡪 PC breach | -.50 | .22 | .03 | Not used to test hypotheses |
| Job demands 🡪 Positive affect 🡪 PC breach | -.20 | .41 | .49 | Not used to test hypotheses |
| Job demands 🡪 Negative affect 🡪 PC breach | 1.74 | .59 | .003 | Hypothesis 6 supported |

*Figure 10. Estimates obtained in the partial mediation model with momentary relationships in Study 2.*

**Time-lagged relationships.** We re-estimated the same mediation model, but this time using time-lagged variables. This means that we estimated paths from an independent variable measured at time T to a dependent variable measured at time T+1. For example, we estimated if there was a positive relationship between job demands experienced during the present week (T) and negative affect experienced during the subsequent week (T+1). In addition, we controlled for the dependent variable at time T, when predicting that same dependent variable at time T+1. This path represents an autoregressive effect. As a result, we explain change in the dependent variable from one week to another. We used these time-lagged variables for two reasons. First, they allowed us to demonstrate temporal precedence in the relationships. Second, they diminished the influence of common method variance.

Comparing a full mediation model to a partial mediation showed that both fitted equally well to the data (χ^2^(2)=4.79, *p*=.09). We decided to proceed with the partial mediation model to maximize comparability of the results with those obtained in Study 1 (see Figure 11). This partial mediation model fitted adequately to the data, except for the RMSEA and TLI indicators (χ^2^(4)=18.13, RMSEA=.12, CFI=.91, TLI=.35). In addition, the Standardized Root Mean Square Residual was well below the recommended cut-off of .05 at the within- and between-person level (SRMR_within_=.00, SRMR_between_=.04), suggesting that the model fit might be satisfactory overall. Hypotheses 1 and 2 could be confirmed as job resources were positively related to positive affect the subsequent week (γ=.22, *p*<.001) and job demands were positively related to negative affect the subsequent week (γ=.40, *p*=.01). Hypothesis 3 could also be confirmed as positive affect was negatively related to PC breach the subsequent week (β=-8.78, *p*<.001). Hypothesis 4 was rejected as negative affect was not significantly related to PC breach the subsequent week (β=-.11, *p*=.97).

Indirect effects were estimated using the products-of-coefficients approach. The indirect effect of job resources on PC breach, via positive affect was calculated by multiplying the path estimate of the relationship between job resources at time T and positive affect at time T+1 ($\hat{a}=.22$) with the path estimate of the relationship between positive affect at time T and PC breach at time T+1 ($\hat{b}=-8.78$). We found a significant indirect effect of job resources on PC breach via positive emotions (*Point estimate*=-1.95, *p*<.001), lending support to Hypothesis 5. The indirect effect of job demands on PC breach, via negative affect was calculated by multiplying the path estimate of the relationship between job demands at time T and negative affect at time T+1 ($\hat{a}=.40$) with the path estimate of the relationship between negative affect at time T and PC breach at time T+1 ($\hat{b}=.11$). However, we could not find support for an indirect effect of job demands on PC breach via negative affect (*Point estimate*=-.04, *p*=.97), disconfirming Hypothesis 6.

*Table 3. Indirect effects in Study 2 – time-lagged relationships.*

| Indirect effect | Point estimate | *SE* | *p* | Conclusion |
| --- | --- | --- | --- | --- |
| Job resources (T) 🡪 Positive affect (T+1 / T) 🡪 PC breach (T+1) | -1.95 | .40 | .000 | Hypothesis 5 supported |
| Job resources (T) 🡪 Negative affect (T+1 / T) 🡪 PC breach (T+1) | -.04 | .90 | .97 | Not used to test hypotheses |
| Job demands (T) 🡪 Positive affect (T+1 / T) 🡪 PC breach (T+1) | .88 | .51 | .08 | Not used to test hypotheses |
| Job demands (T) 🡪 Negative affect (T+1 / T) 🡪 PC breach (T+1) | .04 | 1.04 | .97 | Hypothesis 6 rejected |


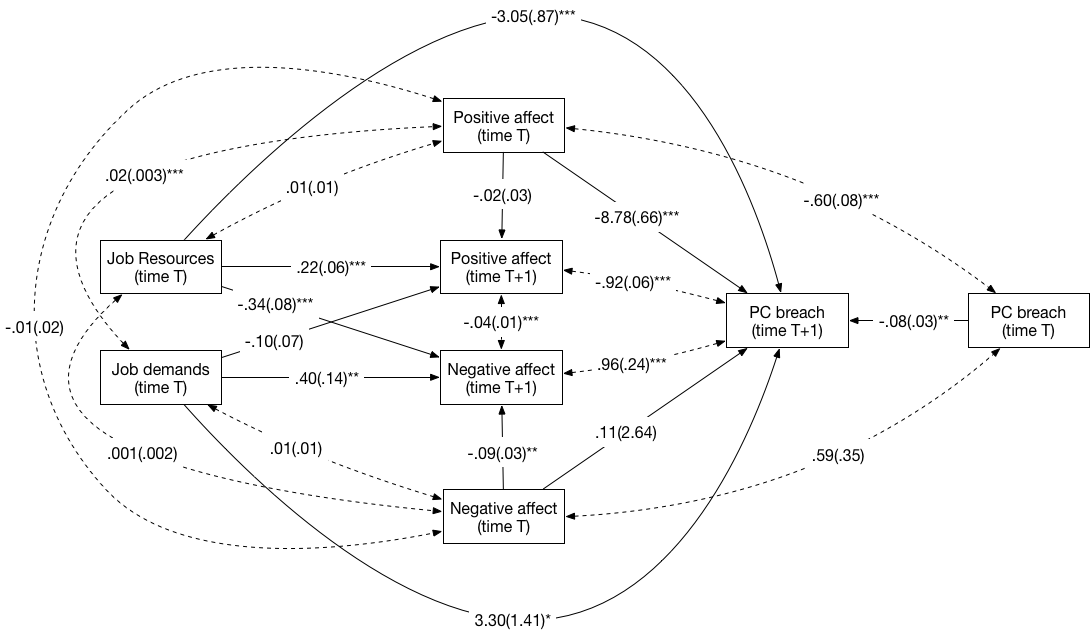


*Figure 11. Path estimates from time-lagged relationships model in Study 2.*

***Sensitivity analyses***

We performed a number of sensitivity analyses to assess if the results obtained in the momentary and time-lagged relationships path models were influenced by third factors. In a first sensitivity analysis, we tested if distinct job demands (cognitive load and workload) and job resources (social support and autonomy) had similar or distinct effects on PC breach, via positive and negative affect. In a second sensitivity analysis, we assessed if the strength of the relationship between job demands and negative affect and the relationship between job resources and positive affect depended on the extent to which respondents expected job demands and resources. For example, it is possible that high job demands only lead to negative affect if respondents do not expect high job demands as part of their job. In a third sensitivity analysis, we examined if the relationships between job demands and resources, positive and negative affect, and PC breach were similar or different for paid employees and volunteers.

**Differences between distinct job demands and resources.** We estimated two path models to test if different job demands and resources had similar or distinct relationships with positive and negative affect (see Figure 12). In the constrained model, the paths from cognitive load and workload to positive and negative affect were constrained to be equal to each other (see Figure 12, left panel). Likewise, the paths from social support and autonomy to positive and negative affect were also constrained to be equal to each other. In the unconstrained model, these paths were freely estimated (see Figure 12, right panel). A χ^2^-difference test showed that the unconstrained model fitted significantly better to the data than the constrained model (χ^2^(4)=43.76, *p*<.001). This implies that different job demands and resources have distinct relationships with positive and negative affect. As can be seen in Figure 12 (right panel), workload at time T-1 was negatively related to positive affect at time T (γ=-.08, *p*=.001) and positively related to negative affect at time T (γ=.13, *p*=.001). Cognitive load at time T-1 was unrelated to positive affect at time T (γ=-.02, *p*=.65), but was positively related to negative affect at time T (γ=.25, *p*<.001). Social support at time T-1 was positively related to positive affect at time T (γ=.36, *p*<.001), and was unrelated to negative affect at time T (γ=-.16, *p*=.07). Autonomy at time T-1 was unrelated to positive (γ=-.12, *p*=.09) and negative affect (γ=-.09, *p*=.26) at time T. Inspection of standardized parameter estimates showed that of the two job demands, cognitive load (B=.15) was more strongly related to negative affect than workload (B=.09); of the two job resources, social support (B=.30) was more strongly related to positive affect than autonomy (B=-.09). We also estimated indirect effects for the different job demands and resources, using the product-of-coefficients approach. There was an indirect effect of workload on PC breach via positive affect (*Point estimate*=.92, *p*=.01) and an indirect effect of social support on PC breach via positive affect (*Point estimate*=-3.96, *p*<.001).


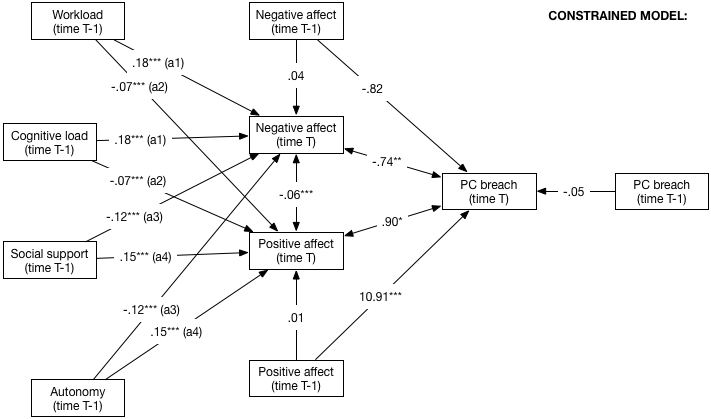

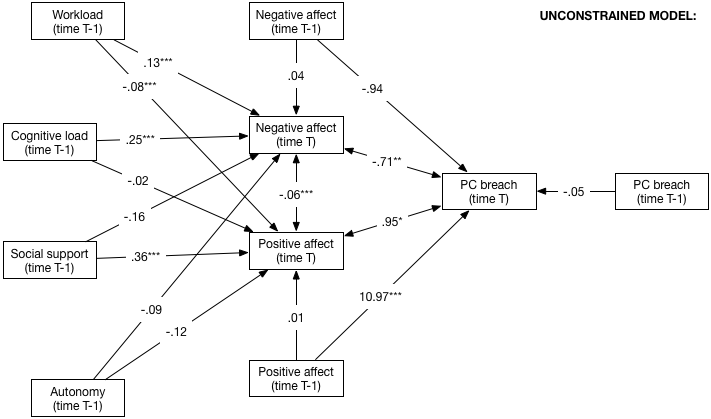


*Figure 12. Constrained and unconstrained model used to assess differences between distinct job demands and resources in Study 2.*

**Role of expected job demands and resources.** We assessed if expectations regarding the amount of job demands or job resources would moderate the relationships between actual job demands and resources on the one hand and positive and negative affect on the other. To this end, we estimated multilevel moderated mediation models using a Bayesian estimator in Mplus version 7, in which expected job demands or job resources served as cross-level moderators. We resorted to Bayesian methods due to convergence issues when using traditional maximum likelihood methods; Bayesian methods allow for estimating complex models when traditional estimation methods fail [6]. An important difference between Bayesian methods and traditional frequentist approaches is that Bayesian methods result in a posterior probability ($P(\Theta|z)$), which represents the probability of an estimated parameter *after* observing the data. In contrast, frequentist approaches yield *p*-values that represent the probability of an estimated effect, assuming that there is no effect in the population. To arrive at this *p*-value, frequentist approaches “rely on hypothetical infinite replications of the Study (via sampling distributions) that never occurred” [6, p. 393]. To interpret estimates in Bayesian analysis, one uses *credibility intervals*. For example, a 95% credibility interval ranging from .10 to .30 means that there is a 95% chance that the estimate lays between .10 and .30. The estimate is significant if zero is not included in the credibility interval. For a detailed explanation of Bayesian methods, we refer the reader to Kruschke, Aguinis and Joo [7] and Zyphur and Oswald [6].

We estimated four models (see Figure 13), to test if the extent to which job demands or job resources were expected moderated the relationship between job demands and resources, and positive and negative affect. We followed recommendations by Preacher, Zyphur, and Zhang [8] and estimated 1-1-1 mediation models with random slopes. This means that the independent variable (e.g., job demands), mediator variable (e.g., negative affect), and dependent variable (e.g., PC breach) were all situated at the within-person level. The paths between the independent and the mediator variable, between the independent and the dependent variable, and between the mediator and the dependent variable were allowed to vary between persons (i.e., random slopes). At the between-person level, the cross-level moderator (e.g., promised job demands) was allowed to explain variance in the random slope representing the relationship between the independent and the mediator variable. We used non-informative priors when estimating models.

As can be seen in Figure 13 (first panel from the top), the cross-level interaction effect of promised demands on the relationship between job demands and negative affect was not significant, as zero was included in the 95% credibility interval (*estimate*=.-.23, 95% credibility interval=[-.73 to .32]). The cross-level interaction effect of promised demands on the relationship between job demands and negative affect (see Figure 13, second panel from the top) was also not significant (*estimate*=.33, 95% credibility interval=[-.09 to .76]). The cross-level interaction effect of promised resources on the relationship between job resources and negative affect (see Figure 13, third panel from the top) was also not significant (*estimate*=-.11, 95% credibility interval=[-1.73 to .32]). The cross-level interaction effect of promised resources on the relationship between job resources and positive affect (see Figure 13, bottom panel) was again not significant (*estimate*=-.01, 95% credibility interval=[-1.05 to .92]). Hence, we can conclude that the extent to which job demands and resources were expected did not moderate the relationships between actual job demands and resources and positive and negative affect.

**
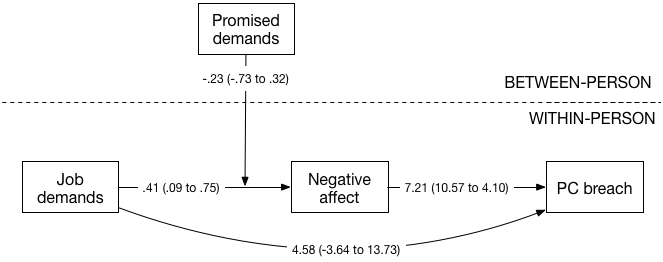

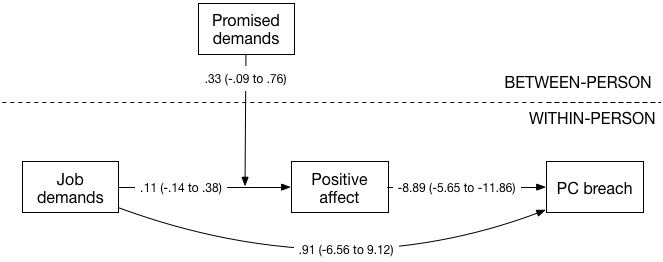

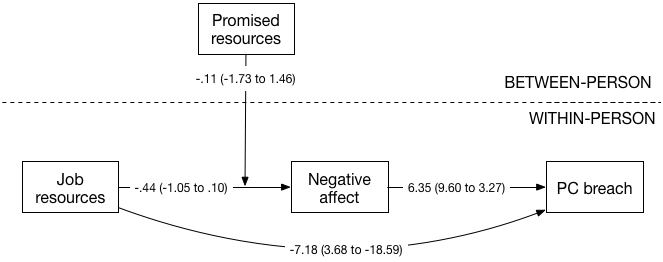

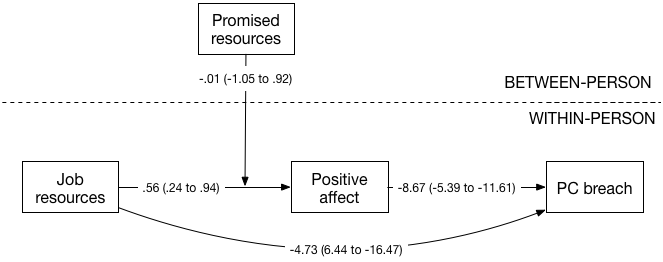
**

*Figure 13. Models used to evaluate cross-level moderating effect of promised job demands and resources (credibility intervals between parentheses).*

**Differences between volunteers and paid employees.** We tested if the estimated relationships in our path analysis model differed between volunteers and paid employees. We therefore performed a multiple-group path analysis and tested two versions of the main research model with time-lagged variables. In the constrained model, the relationships between job demands, job resources, positive affect, negative affect, and PC breach were constrained to be equal for volunteers and paid employees. In the unconstrained model, these relationships were freely estimated in the volunteer and paid employee groups, meaning that relationships were allowed to differ between both groups. A χ^2^-difference test showed that the constrained model fitted equally well to the data as the unconstrained model (χ^2^(24)=32.23, *p*=.12). When two models fit equally well to the data, the simpler, more parsimonious model (i.e., the constrained model) is to be preferred. We therefore conclude that volunteers and paid employees react similarly to job demands and resources, in terms of positive and negative affect and PC breach.

**References**

1. Dyer NG, Hanges PJ, Hall RJ (2005) Applying multilevel confirmatory factor analysis techniques to the Study of leadership. Leadersh Q 16: 149–167. doi:10.1016/j.leaqua.2004.09.009.
2. Schermelleh-Engel K, Moosbrugger H, Müller H (2003) Evaluating the fit of structural equation models: Tests of significance and descriptive goodness-of-fit measures. Methods Psychol Res Online 8: 23–74.
3. Stevens J (2002) Applied multivariate statistics for the social sciences. 4th ed. Mahwah, NJ: Erlbaum.
4. Podsakoff PM, MacKenzie SB, Lee J-Y, Podsakoff NP (2003) Common method biases in behavioral research: A critical review of the literature and recommended remedies. J Appl Psychol 88: 879–903. doi:10.1037/0021-9010.88.5.879.
5. Byrne B (2011) Structural equation modeling with Mplus: Basic concepts, applications, and programming. New York: Routledge.
6. Zyphur MJ, Oswald FL (2013) Bayesian Estimation and Inference: A User’s Guide. 390-420 p.
7. Kruschke JK, Aguinis H, Joo H (2012) The Time Has Come: Bayesian Methods for Data Analysis in the Organizational Sciences. Organ Res Methods 15: 722–752.
8. Preacher KJ, Zyphur MJ, Zhang Z (2010) A general multilevel SEM framework for assessing multilevel mediation. Psychol Methods 15: 209–233.
